# Validation of the Warwick-Edinburgh Mental Well-Being Scale for the Mental Health Surveillance (MHS) of German adults

**DOI:** 10.1186/s12955-024-02304-4

**Published:** 2024-10-26

**Authors:** Diana Peitz, Julia Thom, Lena Walther, Heike Hoelling, Caroline Cohrdes

**Affiliations:** Department of Epidemiology and Health Monitoring, Robert Koch Institute, Berlin, Germany

**Keywords:** Mental Well-Being, WEMWBS, Positive Mental Health, Public Mental Health, Mental Health Surveillance (MHS), Surveillance, Monitoring, Validation, Psychometric Properties, Adults

## Abstract

**Background:**

Mental health encompasses more than just the absence of mental disorders. Thus, a Mental Health Surveillance (MHS) and reporting system for Germany should monitor mental well-being in addition to psychopathology to capture a more complete picture of population mental health. The Warwick-Edinburgh Mental Well-Being Scale (WEMWBS) is an internationally established inventory for the integrated assessment of different aspects of mental well-being (i.e., hedonic and eudaimonic) in population samples that has not yet been validated for Germany.

**Methods:**

Using data from a cross-sectional online survey of a convenience sample of *N* = 1.048 adults aged 18–79 years (51% female) living in Germany, the factorial structure, measurement invariance (age, sex) and psychometric properties of the WEMWBS in its long (14 items) and short (7 items) versions were analyzed. Additionally, correlations to relevant factors (e.g., health-related quality of life, psychological distress) were investigated as indicators of criterion validity.

**Results:**

Means of model fit indices did not confirm a unidimensional factor structure for either version. The three-factor-correlative models showed moderate to good fit while the bifactor model with one general mental well-being factor and three grouping factors fitted the data best. The full range of possible responses was used for all items, and the distribution of both scales was approximately normal. Moreover, the results revealed measurement invariance across sex and age groups. Initial evidence of criterion validity was obtained. Internal consistencies were α = 0.95 and α = 0.89, respectively. Average mental well-being was comparable to that of other European countries at 3.74 for the long version and 3.84 for the short version. While there were no differences by sex, comparisons between age groups revealed higher mental well-being among the older age groups.

**Conclusions:**

Both versions of the WEMWBS showed sound psychometric characteristics in the present German sample. The findings indicate that the instrument is suitable for measuring mental well-being at the population level due to its distributional properties. These results are promising, suggesting that the scale is suitable for use in a national MHS that aims to capture positive mental health in the population as a foundation for prevention and promotion efforts within public mental health.

**Supplementary Information:**

The online version contains supplementary material available at 10.1186/s12955-024-02304-4.

## Background

The regular and systematic surveillance of population health including mental health is crucial for effective public health practice. Surveillance aims to monitor the current state of population mental health and to evaluate trends. The data it provides enables practitioners and political stakeholders to plan and evaluate public mental health measures as well as to respond rapidly and effectively to potentially adverse effects caused by crisis [1, 2].

The German Mental Health Surveillance (MHS) was established at the Robert Koch Institute (RKI) in 2019 and has since been in ongoing development. The MHS is intended to regularly collect, integrate, process, analyze and interpret data on the mental health of the population. Timely reporting of findings on the current state and trends of population mental health is to provide a reliable database for evidence-based policy [3, 4]. To develop an MHS for Germany, suitable core indicators were identified within a structured consensus building process involving experts and stakeholders. In accordance with the dual continua model of mental health [2], both indicators capturing psychopathology in the population and indicators of positive mental health were included in the final set.

The dual continua model of mental health assumes that psychopathology (i.e., represented by mental disorders) and positive mental health (i.e., represented by mental well-being) are two related but distinct dimensions comprising shared as well as distinct predictors (see [5] for an overview). This assumption is in line with the definition of good mental health encompassing more than the absence of mental disorders [6]. With regard to monitoring public mental health, information on the dimension of psychopathology (i.e., the spectrum ranging from psychological distress to mental disorders) is particularly important for initiating and evaluating public health measures addressing mental health care and rehabilitation. Data on positive mental health (including mental well-being) provides information on the need for mental health promotion and prevention [2]. Therefore, mental well-being should be assessed and reported as a distinct dimension (and with its own measurement instrument) in addition to psychopathology in an MHS for Germany.

The WHO defines good mental health as a “a state of well-being in which the individual realizes his or her own abilities, can cope with the normal stresses of life, can work productively and fruitfully, and is able to make a contribution to his or her community” [6]. In line with this definition and from other reviews on well-being, we refer to *mental well-being* as a generic term different from physical well-being and encompassing components of *hedonic* (“feeling good”) and *eudaimonic* (“functioning well”) well-being, as well as social aspects of well-being (e.g., satisfactory interpersonal relationships) [7–9].

The hedonic perspective focuses on the subjective experience of happiness (high positive affect combined with less negative affect; the so-called *affective-evaluative component* of hedonic well-being) and life satisfaction (the so-called *cognitive-evaluative component* of hedonic well-being) [7]. In the past, hedonic well-being, following Diener, has also been referred to as ‘*subjective well-being’* (defined as “a person’s cognitive and affective evaluation of life” [10]) or in line with Ryff and Keyes ‘*emotional well-being*’ [11].

The eudaimonic perspective focuses on the psychological functioning of a person, including concepts of autonomy, competence, self-acceptance and personal growth as well as positive interpersonal relationships. Eudaimonic well-being has sometimes been referred to as ‘*psychological well-being*’ [11] and was extended by a concept called ‘*social well-being*’, including dimensions of social functioning and relatedness with respect to society [8].

The Warwick-Edinburgh Mental Well-Being Scale (WEMWBS) is an internationally established short measurement tool designed to integratively assess different dimensions of mental well-being on a population level. The WEMWBS covers hedonic aspects with a focus on positive affect (optimism, cheerfulness, relaxation), eudaimonic aspects of psychological functioning (autonomy, competency, self-acceptance, personal growth), and positive interpersonal relationships [12].

The original version with 14 positively worded items was developed in the United Kingdom (UK) and has been translated into various other languages to monitor the mental well-being of the population and various subgroups [13]. Across several countries and populations, the WEMWBS showed psychometrically sound properties such as internal consistency [14], factorial validity [15] and criterion validity regarding a broad range of outcomes (e.g., health-related quality of life [16, 17], psychological distress [18] and psychological resources [12]). These findings suggest that the scale is an appropriate tool for its original purpose of monitoring population mental well-being and evaluating public health promotion and prevention measures. While several validation studies have confirmed the one-factor solution of the original version (e.g., [12]), some validation studies provide evidence suggesting three key factors as originally intended by the expert panel involved in the scale’s construction (i.e., hedonic, eudaimonic, interpersonal relationships [17, 19–21]). Recent evidence shows, that the factor structure might be best explained by applying a bifactor model with one general mental well-being factor and three grouping factors representing the structure mentioned above [20–22].

Concerns about item redundancy led to the construction of a brief 7-item version by the authors of the original scale using Rasch models. Although this short version (SWEMWBS) prioritizes the eudaimonic perspective over the hedonic perspective, its use is recommended in population surveys due to its brevity and robust psychometric properties [23].

There is a German version of the WEMWBS from Austria validated in an Austrian sample [17], but neither a long version nor a short version has been validated in a German sample. Hence, the present study aimed to validate this German version of the (S)WEMWBS and assess its suitability as the measure of mental well-being for the German MHS.

## Materials and methods

### Procedure and participants

The sample recruitment and data collection was conducted by a market and opinion research agency (respondi AG) on behalf of the RKI. Participants were recruited via the in-house access panel (convenience sample). Data were collected by means of a cross-sectional online survey in December 2020. After providing informed consent, participants answered several questions on socioeconomic (e.g., age, sex, education [24], employment status) and health characteristics (e.g., self-reported diagnoses of chronic conditions and/or lifetime mental disorders [*Have you ever been diagnosed with a chronic condition/mental disorder in your life?]*). The forced choice character of the items allowed for analyses using the full data set. In total, *N* = 1.048 participated in the study, balanced by sex (female/male) and age group (18–34, 35–49, 50–64, 65–79 years). Further sample characteristics are shown in Table 1.

**Table 1:** Sample characteristics

Characteristic	Category	*n*	%
Total		1048	100
Sex	female	531	51
	male	517	49
Age group	18–34 years	258	25
	35–49 years	259	25
	50–64 years	267	25
	65–79 years	264	25
Education (CASMIN)^1^	Low	117	11
	Moderate	370	35
	High	556	53
Employment status	full time	436	42
	part time	162	15
	unemployed	450	43
Partnership status	partnered	633	60
	single	415	40
Chronic disease^2^	Yes	484	47
	No	537	53
Mental disorder^2^	Yes	263	25
	No	769	75

Note.^1^ = in accordance with Comparative Analyses of Social Mobility in Industrial Nations; CASMIN (24). ^2^ = self-reported lifetime diagnosis

## Measures

### Warwick-Edinburgh mental well-being scale

Participants completed the German translation of the 14-item WEMWBS [17] on a 5-point scale (1 = ‘none of the time’ to 5 = ‘all of the time’) referring to a two-week period. The total score ranges from 14 to 70, with higher scores indicating higher mental well-being. Participants’ responses to the long version were also used to validate the 7-item short version (with a total score ranging from 7 to 35). Items and item characteristics of the (S)WEMWBS are presented in Table 2.

**Table 2:** (S)WEMWBS: item statistics (*N* = 1,048, 18–79 years)

Items (in serial order)						Answer Category (total number / percent)				
		Mean	SD	Skewness	Kurtosis	1	2	3	4	5
*#1*	*I’ve been feeling optimistic about the future.* ^*h*^	3.47	1.03	|0.42|	|0.36|	40	146	299	406	157
	*Ich habe mich in Bezug auf die Zukunft optimistisch gefühlt.*					4%	14%	28%	39%	15%
*#2*	*I’ve been feeling useful.* ^*i*^	3.70	0.97	|0.57|	|0.03|	23	98	264	452	211
	*Ich habe mich nützlich gefühlt.*					2%	10%	25%	43%	20%
*#3*	*I’ve been feeling relaxed.* ^*h*^	3.60	0.91	|0.53|	|0.09|	21	101	298	480	148
	*Ich habe mich entspannt gefühlt.*					2%	10%	28%	46%	14%
#4	I’ve been feeling interested in other people.^i^	3.74	0.90	|0.52|	|0.14|	15	71	285	474	203
	Ich habe mich für andere Menschen interessiert.					1%	7%	27%	45%	20%
#5	I’ve had energy to spare.^i^	3.40	0.96	|0.42|	|0.26|	34	155	323	433	103
	Ich hatte viel Energie.					3%	15%	31%	41%	10%
*#6*	*I’ve been dealing with problems well.* ^*e*^	3.94	0.81	|0.65|	|0.66|	9	35	220	531	253
	*Ich bin mit Problemen gut umgegangen.*					1%	3%	21%	51%	24%
*#7*	*I’ve been thinking clearly.* ^*e*^	4.30	0.79	|1.05|	|0.92|	3	28	114	412	491
	*Ich konnte klar denken.*					1%	3%	11%	39%	47%
#8	I’ve been feeling good about myself. ^*h*^	3.75	0.86	|0.54|	|0.25|	12	68	276	505	187
	Ich habe mich wohl gefühlt.					1%	7%	26%	48%	18%
*#9*	*I’ve been feeling close to other people.* ^*i*^	3.58	1.02	|0.51|	|0.23|	34	123	278	425	188
	*Ich habe mich anderen Menschen nahe gefühlt.*					3%	12%	27%	40%	18%
#10	I’ve been feeling confident. ^*h*^	3.67	0.91	|0.52|	|0.03|	17	95	284	477	175
	Ich habe mich zuversichtlich gefühlt.					2%	9%	27%	45%	17%
*#11*	*I’ve been able to make up my own mind about things.* ^*e*^	4.26	0.82	|1.02|	|0.88|	6	25	140	400	477
	*Ich war in der Lage*,* Entscheidungen zu treffen.*					1%	2%	13%	38%	46%
#12	I’ve been feeling loved. ^*i*^	3.76	1.05	|0.64|	|0.17|	34	94	251	379	290
	Ich habe mich geliebt gefühlt.					3%	9%	24%	36%	28%
#13	I’ve been interested in new things. ^*e*^	3.62	0.96	|0.39|	|0.27|	20	108	315	411	194
	Ich habe mich für Neues interessiert.					2%	10%	30%	39%	19%
#14	I’ve been feeling cheerful. ^*h*^	3.53	0.91	|0.46|	|0.09|	19	123	312	474	120
	Ich habe mich fröhlich gefühlt.					2%	12%	30%	45%	11%

Note: Table shows original items in English and the German translation (17). Items of the SWEMWBS are marked in italics. SD = standard deviation, h = hedonic aspect, e = eudaimonic aspect, i = interpersonal relationship aspect

To assess the criterion validity of the scale’s short and long versions, associations to related constructs (hedonic well-being, health-related quality of life, psychological distress, proactive coping and self-efficacy) were examined. Hedonic well-being was measured using the WHO-5 (Bech, 2004 [25–27]). Health-related quality of life was measured using the *Short-Form Health Survey (SF-12* [28]), the *Assessment of Quality of Life – 6D* scale (*AQoL-6D* [29]) and the *WHOQoL-BREF*; [30]. Psychological distress was operationalized with the *Kessler-10 distress scale (K-10* [31–33]). Proactive coping was measured using the *Proactive Coping Inventory* (PCI [34, 35]), and self-efficacy was assessed with the *Allgemeine Selbstwirksamkeitsskala* (ASKU [36]). Detailed information on these instruments is included in the supplementary materials. In accordance with findings from other validation studies [12, 15–17], we expected high convergent validity with instruments assessing closer constructs, namely, *hedonic well-being*, health-related *quality of life* (positive correlations) and *psychological distress* (negative correlation). Small to moderate positive associations were expected for the instruments assessing related but more distant constructs reflecting psychological resources, namely, *proactive coping* and *self-efficacy*.

### Statistical analyses

The analyses were carried out with the software *R statistics* (R Version 4.4.0; RStudio Version 2024.04.0 + 735 “Chocolate Cosmos” 3) [37] in the following five steps: testing (1) factorial validity and (2) measurement invariance as well as (3) criterion validity, (4) face validity, and (5) internal consistency.

### Factorial validity

To test the factorial structures of the (S)WEMWBS for different models, confirmatory factor analyses (CFA) were performed using *maximum likelihood parameter estimates using standard errors robust to non-normality* (MLR) and computed by means of the *R* package ‘*lavaan*’ [38]. The chi-square (*χ*2) value is highly affected by the size of the sample [39]. Thus, the standardized root mean square residual (SRMR), the root mean square error of approximation (RMSEA) and the comparative fit index (CFI) were used to evaluate the model fit [40]. Cutoffs for an acceptable fit were set as RMSEA ≤ 0.08, SRMR ≤ 0.10 and CFI ≥ 0.95, while a good model fit was assumed with RMSEA ≤ 0.05, SRMR ≤ 0.05 and CFI ≥ 0.97 [41]. Fit indices were compared to explore whether the factorial structure could be better explained by the suggestions of the one factor model published in the original UK validation [12] or the three factor model presented by the Austrian validation [17], respectively.

Since evidence for the appropriateness of fitting the (S)WEMWBS has accumulated recently (e.g [20–22]). and the Austrian validation based on a German-speaking sample used the same modelling [17], we additionally calculated a bifactor model as suggested by Eid and colleagues [42]. The bifactor-(SI – 1) model included item #6 as reference item loading only on the general factor. The selection of the reference item is based on theoretical and statistical considerations based on other data as described in Cohrdes and Junker [21].

### Measurement invariance

Measurement invariance was investigated for self-reported sex (female and male) and age (above and below the sample’s mean of 50 years following the approach of Koushede and colleagues [15]). The following three measurement invariance steps were taken into account: (1) configural (equivalence of model form), (2) metric (equivalence of factor loading), (3) scalar (equivalence of item intercepts) [40]. Differences for Δ RMSEA ≤ 0.015, Δ CFI ≤ 0.010 and Δ SRMR ≤ 0.030 from configural to metric invariance and Δ RMSEA ≤ 0.015, Δ CFI ≤ 0.010 and Δ SRMR ≤ 0.015 from metric to scalar invariance were considered acceptable [43].

Measurement invariance was tested with the ‘*semTools*’ package and the *measEq.syntax* command using theta-parametrization and by standardizing the common factor [44]; model comparisons were performed with the *comparFit* command from *‘semTools’*. For measurement invariance we followed the procedure as recommended by Jorgensen and colleagues [44] and fitted one model at a time. After establishing invariance of thresholds, we proceeded to test equivalence of loadings and intercepts (metric and scalar invariance, respectively).

### Criterion validity

Pearson product-moment correlations were used to estimate the associations between the (S)WEMWBS and relevant external criteria as indicators of criterion (convergent) validity. In accordance with Cohen [45], the strength of association was interpreted as follows: *r >* .10 small, *r >* .30 medium, *r >* .50 large effect size.

### Face validity - descriptive item analysis

Response behavior was checked via frequency analyses for each item to analyze the appropriateness of the WEMWBS 5-point scale. Distributions of single item scores and total scores were analyzed via visual inspection of histograms and residual plots. Frequency analyses were used to assess the mean, standard deviation, skewness (< |2.0|) and kurtosis (< |7.0|). On the basis of these analyses, ceiling and floor effects were checked carefully.

### Internal consistency and Scale properties

Cronbach’s alpha (α) was used as a reliability estimator. Additionally, in congruence with other validations of the (S)WEMWBS, internal consistency estimates greater than *r* = .7 were considered appropriate [14, 19]. Estimates above 0.9 were considered an indicator of item redundancy. Group differences were determined by *t-*tests and the strength of differences was indicated by Cohen’s *d*.

## Results

### Factorial validity

The unidimensional factor structure showed no acceptable fit indices regarding RMSEA and CFI for both versions. For both the short and long scale versions, the correlated three-factor models showed superiority over the one-factor models, as indicated by better fit indices (considered as moderate to good with regard to all applied indices except RMSEA (see Table 3). The bifactor model fitted the data best with fit indices considered as good for both versions except RMSEA for the long version, which indicated moderate fit. The amount of common variance explained for the bifactor models for the WEMWBS were 0.87 (general factor), 0.04 (eudaimonic), 0.17 (hedonic), 0.26 (interpersonal relationships) and for the SWEMWBS 0.78 (general factor), 0.26 (eudaimonic), 0.25 (hedonic), 0.28 (interpersonal relationships). Detailed results of all for models can be find in the Supplementary Materials (Tables S1.1-S1.6; Tables S2.1-S2.6).

Modification indices were considered for the best fitting models (3-factor solutions and bifactor models) in order to explore improvements:

For the the WEMWBS (3-factor solution) based on modification indices we allowed items #2 and #13 to also load on the respective two other factors (#2: eudaimonic, interpersonal relationship; #13: hedonic, interpersonal relationship). This resulted in an improved model fit (*X*^*2*^_*(72)*_ = 554.740, *p* < .001, CFI = 0.956, RMSEA = 0.078 CI [0.071–0.085], SRMR = 0.044, AIC = 29079.056.) However, modification indices of this modified model suggested further model improvement by removing either item #7 or #11 due to high intercorrelatedness (MI = 119.296). The WEMWBS bifactor model consistently showed potential for model improvement by allowing item #2 to also load on the hedonic and interpersonal relationship factor based on modification indices (MI = 39.307, 35.574, respectively). Moreover, item #13 revealed a negative loading on the eudaimonic factor. The fit indices for a modified model allowing item #2 to also load on the other two factors and excluding item #13 improved with *X*^*2*^_*(49)*_ = 236.539, *p* < .001, CFI = 0.982, RMSEA = 0.058 CI [0.049–0.067], SRMR = 0.019, AIC = 26477.716.

Based on the modification indices of the 3-factor solution of the SWEMWBS we allowed item #6 to also load on the other factors (hedonic, interpersonal relationship). This resulted in an improved model fit of (*X*^*2*^_*(9)*_ = 49.250, *p* < .001, CFI = 0.990, RMSEA = 0.063 CI [0.043–0.085], SRMR = 0.020, AIC = 15736.330). For the SWEMWBS bifactor model, modification indices showed no potential for model improvement.

**Table 3:** Model fit indices resulting from CFA using maximum-likelihood estimation with robust standard errors

Model	*χ* ^2^_(df)_	RMSEA (90%CI)*	CFI	SRMR	AIC
**WEMWBS**					
One-factor model	1063.220_(77)*_	0.109 (0.102–0.116)	0.909	0.053	29577.536
Three-factor model	637.344_(74)*_	0.083 (0.076–0.090)	0.949	0.046	29157.660
Bifactor model	339.882_(61)*_	0.064 (0.056–0.072)	0.975	0.025	28886.198
**SWEMWBS**					
One-factor model	348.084_(14)*_	0.149 (0.133–0.166)	0.909	0.059	16025.163
Three-factor model	122.645_(11)*_	0.097 (0.079–0.116)	0.970	0.036	15805.724
Bifactor model	13.845_(5)*_	0.039 (0.008–0.068)	0.998	0.008	15708.925

*Note. N* = 1048. CFA = Confirmatory Factor Analyses, WEMWBS = Warwick-Edinburgh Mental Well-Being Scale, SWEMWBS = Warwick-Edinburgh Mental Well-Being Scale – Short Form. *χ*^2^  = chi-square, RMSEA = robust root mean square error of approximation, 90% CI = 90% confidence interval, CFI = robust comparative fit index, SRMR = standardized root mean square residual, AIC = Akaike information criterion. * *p* < .001

### Measurement invariance

Across sex and age groups and for all tested models (one-factor model, three-factor model, bifactor model), the configural and metric invariance models indicated invariance of model form and factor loadings for both the long and short versions. The change in fit indices between the scalar invariance models and the metric invariance models all met the applied cutoff indices for both versions (WEMWBS and SWEMWBS) in all tested models (one-factor model, three-factor model, bifactor model) for invariant intercepts as well. All values are depicted in Tables 4 and 5.

**Table 4:** Results from measurement invariance analyses across sex groups (male, female) based on maximum-likelihood estimation with robust standard errors (MLR)

Model	X^2^_(df)_	RMSEA (90%CI)*	CFI	SRMR	AIC
**One-factor model** **WEMWBS**					
Configural	1161.6 _(154)_	0.109 (0.102–0.116)	0.909	0.051	29567.853
Metric	1170.7 _(167)_	0.104 (0.097–0.111)	0.910	0.054	29550.903
DIFF Δ	8.501	0.005 (0.004–0.005)	0.000	0.003	16.951
Scalar	1259.2 _(180)_	0.104 (0.098–0.111)	0.902	0.058	29613.445
DIFF Δ	88.311	0.000 (0.000–0.000)	0.007	0.004	62.543
**Three-factor model** **WEMWBS**					
Configural	746.71_(148)_	0.084 (0.077–0.092)	0.948	0.045	29164.923
Metric	758.92_(159)_	0.081 (0.074–0.088)	0.948	0.048	29155.133
DIFF Δ	11.155	0.003 (0.003–0.003)	0.000	0.003	-9.790
Scalar	793.45_(170)_	0.080 (0.073–0.087)	0.080	0.050	29167.661
DIFF Δ	34.325	0.001 (0.001–0.001)	0.002	0.002	12.528
**Bifactor model** **WEMWBS**					
Configural	438.66_(122)_	0.068 (0.060–0.076)	0.972	0.026	28908.875
Metric	463.44_(145)_	0.061 (0.054–0.069)	0.973	0.035	28887.653
DIFF Δ	15.351	0.007 (0.007–0.007)	0.001	0.010	21.222
Scalar	491.15_(155)_	0.061 (0.053–0.068)	0.971	0.037	28895.364
DIFF Δ	24.356	0.000 (0.000–0.001)	0.002	0.002	7.711
**One-factor model** **SWEMWBS**					
Configural	355.72_(28)_	0.147 (0.130–0.164)	0.913	0.053	16014.802
Metric	364.38_(34)_	0.133 (0.119–0.149)	0.912	0.058	16011.460
DIFF Δ	7.590	0.013 (-0.015 - -0.011)	0.001	0.005	3.342
Scalar	408.10_(40)_	0.130 (0.117–0.144)	0.902	0.063	16043.185
DIFF Δ	44.256	0.003 (-0.005 - -0.002)	0.010	0.005	31.725
**Three-factor model** **SWEMWBS**					
Configural	137.62_(22)_	0.097 (0.079–0.116)	0.970	0.034	15808.697
Metric	142.09_(26)_	0.089 (0.072–0.107)	0.970	0.037	15805.169
DIFF Δ	3.4181	0.008 (0.009–0.007)	0.000	0.004	3.528
Scalar	148.36_(30)_	0.084 (0.068–0.100)	0.969	0.038	15803.444
DIFF Δ	6.2627	0.005 (0.007–0.004)	0.001	0.001	1.725
**Bifactor model** **SWEMWBS**					
Configural	14.788_(10)_	0.020 (0.000–0.059)	0.999	0.008	15709.868
Metric	34.161_(19)_	0.037 (0.011–0.059)	0.996	0.025	15711.241
DIFF Δ	22.600*	0.017 (0.000–0.011)	0.003	0.018	1.373
Scalar	43.624_(22)_	0.040 (0.017–0.061)	0.995	0.026	15714.704
DIFF Δ	6.104	0.003 (0.002–0.006)	0.001	0.001	3.463

*Note. N* = 1048. CFA = Confirmatory Factor Analyses, WEMWBS = Warwick-Edinburgh Mental Well-Being Scale, SWEMWBS = Warwick-Edinburgh Mental Well-Being Scale – Short Form. *χ*^2^  = chi-square, RMSEA = robust root mean square error of approximation, 90% CI = 90% confidence interval, CFI = robust comparative fit index, SRMR = standardized root mean square residual, AIC = Akaike information criterion, DIFF = difference. * *p* < .001

**Table 5:** Results from measurement invariance analyses across age groups (above and below the mean value of 50 years) based on maximum-likelihood estimation with robust standard errors

Model	X^2^_(df)_	RMSEA (90%CI)*	CFI	SRMR	AIC
**One-factor model** **WEMWBS**					
Configural	1156.3_(154)_	0.108 (0.101–0.116)	0.909	0.052	29433.667
Metric	1177.2_(167)_	0.104 (0.098–0.111)	0.908	0.059	29428.553
DIFF Δ	19.72	0.004 (0.004–0.004)	0.001	0.007	5.114
Scalar	1323.8_(180)_	0.107 (0.101–0.114)	0.895	0.063	29549.151
DIFF Δ	145.52	0.003 (0.003–0.003)	0.013	0.006	120.597
**Three-factor model** **WEMWBS**					
Configural	727.10_(148)_	0.083 (0.075–0.090)	0.949	0.044	29016.448
Metric	741.56_(159)_	0.080 (0.073–0.087)	0.949	0.049	29008.909
DIFF Δ	12.977	0.003 (0.003–0.002)	0.000	0.004	7.539
Scalar	812.95_(170)_	0.082 (0.075–0.088)	0.939	0.052	29058.296
DIFF Δ	71.477	0.002 (0.001–0.002)	0.006	0.002	49.387
**Bifactor model** **WEMWBS**					
Configural	423.95_(122)_	0.067 (0.060–0.075)	0.972	0.027	28765.291
Metric	455.11_(145)_	0.060 (0.052–0.068)	0.974	0.035	28750.457
DIFF Δ	15.457	0.007 (0.007–0.007)	0.001	0.009	14.834
Scalar	501.51_(155)_	0.062 (0.055–0.069)	0.970	0.037	28776.857
DIFF Δ	45.504	0.002 (0.001–0.002)	0.003	0.002	26.400
**One-factor model** **SWEMWBS**					
Configural	364.46_(28)_	0.149 (0.133–0.166)	0.908	0.054	15902.625
Metric	373.41_(34)_	0.136 (0.121–0.151)	0.908	0.600	15899.567
DIFF Δ	7.643	0.013 (0.012–0.015)	0.001	0.006	3.058
Scalar	444.10_(40)_	0.137 (0.124–0.150)	0.889	0.068	15958.263
DIFF Δ	71.873	0.001 (0.003–0.000)	0.018	0.008	58.696
**Three-factor model** **SWEMWBS**					
Configural	132.54_(22)_	0.095 (0.077–0.114)	0.970	0.033	15682.704
Metric	142.47_(26)_	0.089 (0.073–0.107)	0.969	0.041	15684.627
DIFF Δ	7.207	0.006 (0.007–0.005)	0.001	0.007	1.922
Scalar	174.01_(30)_	0.093 (0.078–0.109)	0.961	0.046	15708.174
DIFF Δ	31.428	0.004 (0.002–0.005)	0.008	0.005	23.548
**Bifactor model** **SWEMWBS**					
Configural	17.914_(10)_	0.037 (0.000–0.067)	0.998	0.009	15592.074
Metric	36.994_(19)_	0.040 (0.016–0.062)	0.995	0.028	15593.154
DIFF Δ	17.239	0.004 (0.005–0.016)	0.003	0.019	1.080
Scalar	47.581_(22)_	0.044 (0.022–0.064)	0.994	0.029	15597.741
DIFF Δ	7.0298	0.004 (0.002–0.006)	0.002	0.001	4.587

*Note. N* = 1048. CFA = Confirmatory Factor Analyses, WEMWBS = Warwick-Edinburgh Mental Well-Being Scale, SWEMWBS = Warwick-Edinburgh Mental Well-Being Scale – Short Form. *χ*^2^ = chi-square, RMSEA = robust root mean square error of approximation, 90% CI = 90% confidence interval, CFI = robust comparative fit index, SRMR = standardized root mean square residual, AIC = Akaike information criterion, DIFF = difference. * *p* < .001

### Criterion validity

SWEMWBS was highly correlated with the full WEMWBS (*r* = .97) as well as with a version of the WEMWBS only including items that are not part of the SWEMWBS (*r* = .90). With regard to criterion validity, all associations between the (S)WEMWBS and the validation scales showed in the expected directions (Table 6).

**Table 6:** Correlations of the (S)WEMWBS with relevant constructs as indicators for criterion validity^1^

Construct Instrument (α)			WEMWBS	SWEMWBS
**Mental well-being**				
WHO-5	(0.93)		0.80^*^	0.76^*^
**Psychological Distress**				
K10	(0.95)		− 0.68*	− 0.68*
**Quality of Life**				
AQoL-6D: mental component	(0.87)		0.63*	0.69*
AQoL-6D: physical component	(0.86)		0.55*	0.56*
WHOQoL-BREF: physical component	(0.84)		0.56*	0.54*
WHOQoL-BREF: mental component	(0.86)		0.78*	0.76*
WHOQoL-BREF: social component	(0.71)		0.59*	0.55*
WHOQoL-BREF: environmental component	(0.79)		0.63*	0.62*
SF-12: physical component^2^	(0.85)		0.18*	0.18*
SF-12: mental component^2^	(0.86)		0.43*	0.42*
**Psychological Resources**				
ASKU (self-efficacy)	(0.90)		0.42*	0.44*
PCI (proactive coping)	(0.86)		0.37*	0.35*
**Properties**				
*M (SUM)*			3.74 (52.31)	3.84 (26.85)
*SD (SUM)*			0.71 (9.92)	0.70 (4.92)
*Minimum (SUM)*			1 (14)	1 (7)
*Maximum (SUM)*			5 (70)	5 (35)
*Skewness (SUM)*			- 0.521	- 0.596
*Kurtosis (SUM)*			0.316	0.424
*Cronbach’s Alpha*			0.946	0.887
*MIC*			0.557	0.534

Note. ^1^*N* = 1048. ^2^*n* = 1000. WEMWBS = Warwick-Edinburgh Mental Wellbeing Scale, SWEMWBS = Warwick-Edinburgh Mental Wellbeing Scale – Short Form, K10 = 10-item Kessler Scale, WHO-5 = WHO-5 Well-Being Index, AQoL = Assessment of Quality of Life instruments, WHOQoL-BREF = World Health Organization Quality of Life Assessment Instrument; Brief Version, SF-12 = Short-Form Health Survey, AKSU = Allgemeine Selbstwirksamkeitsskala [engl.: General Self-Efficacy Scale], PCI = Proactive Coping Inventory. *M* = Mean, *SUM* = Sum Mean, *SD* = Standard Deviation, α *=* Cronbach’s Alpha, *MIC* = Mean Interitem-Correlation. * *p* < .001.

### Face validity - descriptive item analysis

**Fig. 1:**
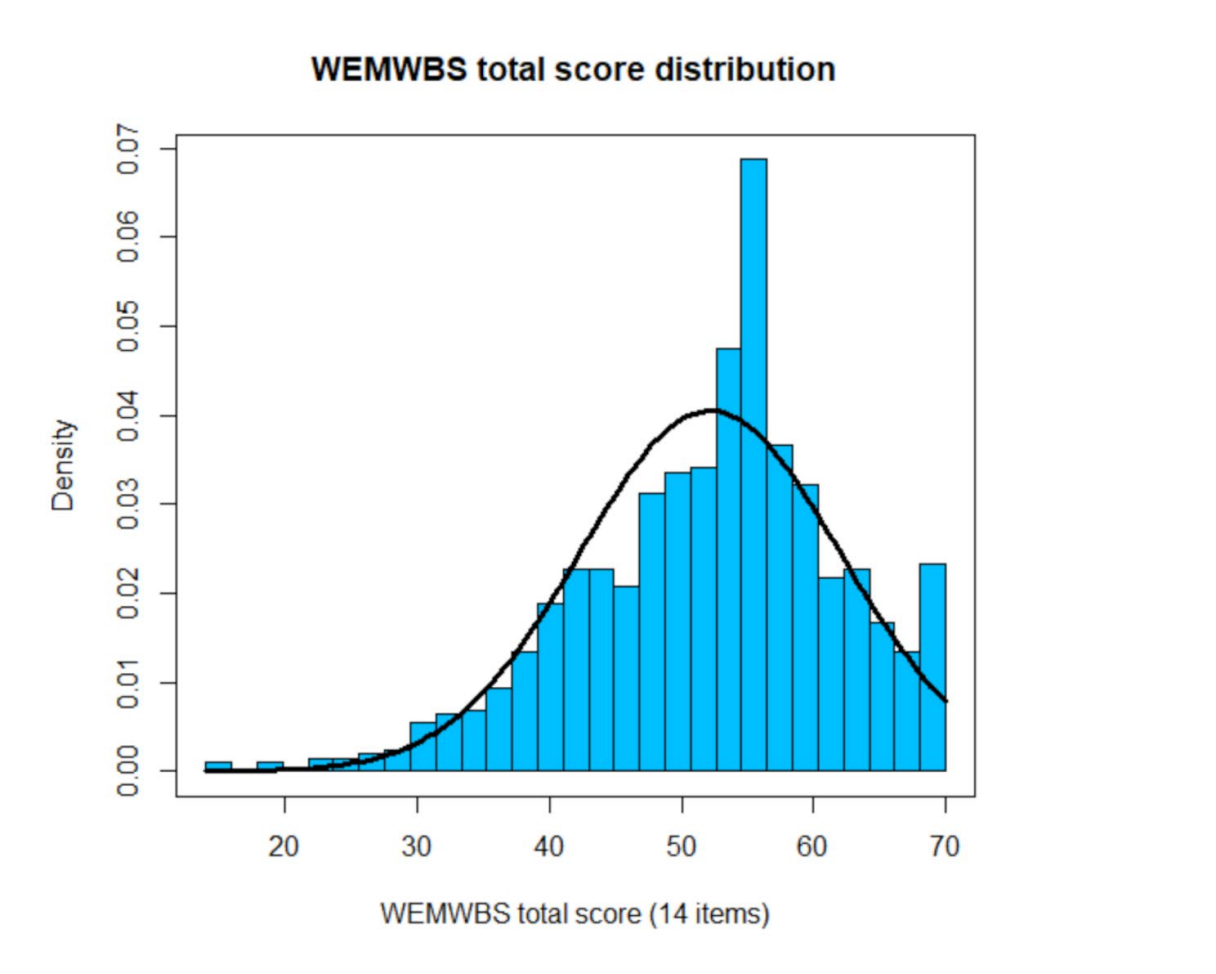
WEMWBS total score distribution

**Fig. 2:**
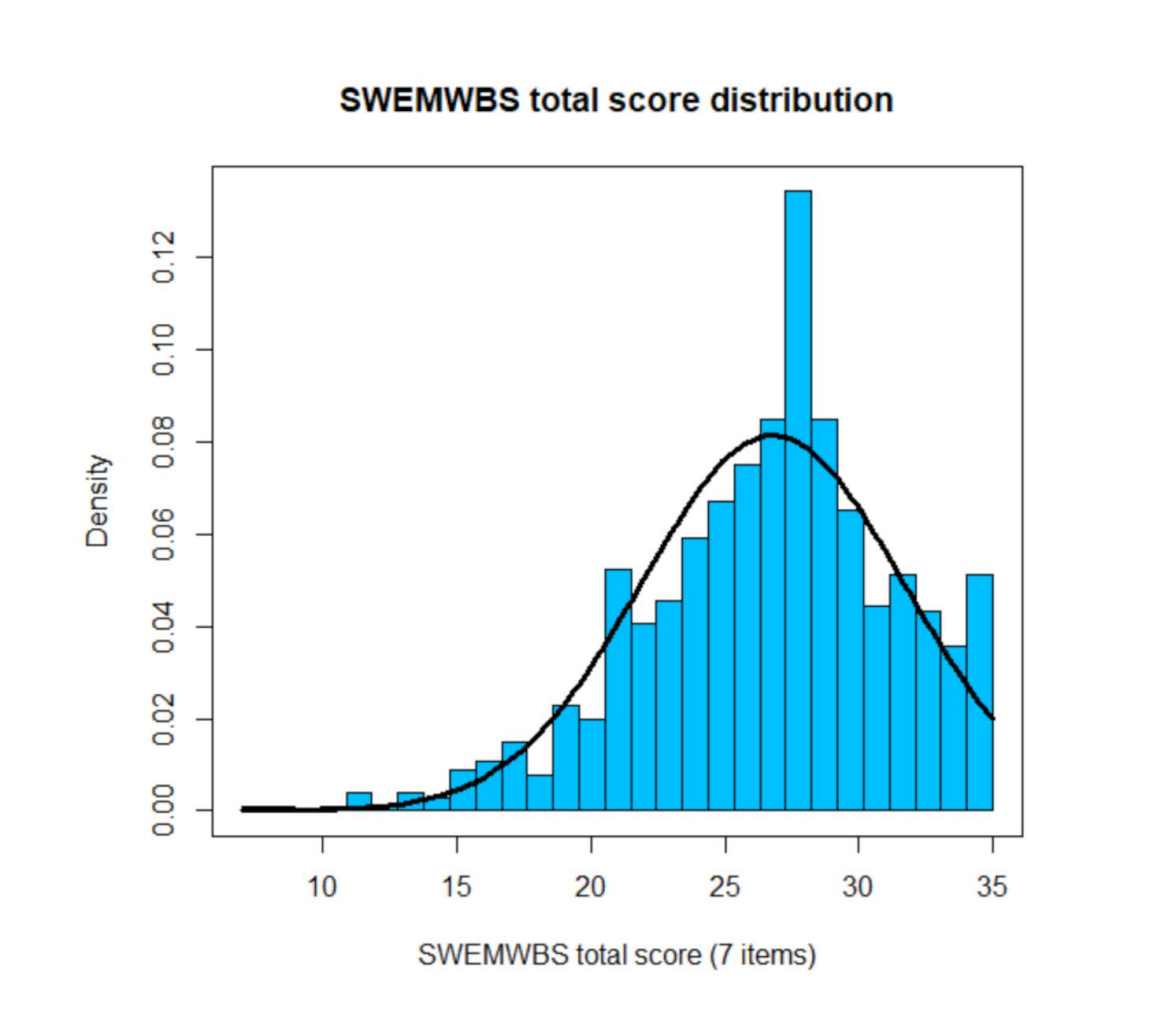
SWEMWBS total score distribution

Table 2 gives an overview of the item means, standard deviations, skewness, kurtosis and response frequencies. The full width of the response format was used with regard to all items. The results indicate appropriateness of the 5-point scale as well as a sufficient differentiation ability of the scale’s items. Figures 1 and 2 show an approximately left-shifted normal distribution of both total scores. No ceiling or floor effects could be detected. Items 7 and 11 showed higher means and smaller variation than the other items. This was found in a different validation study as well [46].

### Internal consistency and scale properties

Cronbach’s alpha and mean well-being scores can be found in Table 6. Higher Cronbach’s alpha values of the WEMWBS compared to the SWEMWBS items indicate item redundancy of the long version and the preferable use of the short version in population surveys, as suggested by the authors of the original versions [12, 20].

There were no significant differences in mental well-being scores (WEMWBS: *t*_(1046)_ = 1.070, *p* = .285, *d* = 0.070; SWEMWBS: *t*_(1046)_ = 0.790, *p* = .430, *d* = 0.043) between males (WEMWBS: *M* = 3.76 (*SD* = 0.70); SWEMWBS: *M* = 3.85 (*SD* = 0.69)) and females (WEMWBS: *M* = 3.71 (*SD* = 0.72); SWEMWBS: *M* = 3.82 (*SD* = 0.71)).

An effect of age could be obtained (WEMWBS: *F*_(1044,3)_ = 8.996, *p* ≤ .001; SWEMWBS: *F*_(1044,3)_ = 11.371, *p* ≤ .001). Post hoc tests (Tukey) revealed that the oldest age group between 65 and 79 years (WEMWBS: *M* = 3.91,*SD* = 0.64; SWEMWBS: *M* = 4.02, *SD* = 0.64) showed significantly higher (*p* ≤ .001) mental well-being than those between 18 and 34 years (WEMWBS: *M* = 3.65,*SD* = 0.73; *d* = 0.370; SWEMWBS: *M* = 3.72, *SD* = 0.73; *d* = 0.44) and those between 35 and 49 years (WEMWBS: *M* = 3.63, *SD* = 0.75,; *d* = 0.40; SWEMWBS: *M* = 3.72,*SD* = 0.73; *d* = 0.44) but not (*p* = .104) higher than those between 50 and 64 years (WEMWBS: *M* = 3.77,*SD* = 0.68; *d* = 0.21; SWEMWBS: *M* = 3.88, *SD* = 0.78; *d* = 0.21).

## Discussion

The assessment of mental well-being is crucial to providing a complete picture of population mental health according to a dual continua model of mental health. Both the long and the short version of the (S)WEMWBS scale have been previously found to be suitable measures of mental well-being and were thus tested in a German sample for their use within Germany’s MHS. In line with other validation studies, the present study finds both versions of the scale to have sufficient psychometric properties.

Means of model fit indices did not confirm a unidimensional factor structure for either version. The three-factor-correlative models were superior to the single-factor models for all chosen cutoffs for the long as well as the short scale, except RMSEA for the SWEMWBS. Interestingly, also other validations of the (S)WEMWBS reported problems with the RMSEA indicating partly insufficient fit while the other used indices showed good fit [20]. However, the bifactor model fitted the data best for both versions with all fit indices indicating sufficient fit. This finding is in line with recent evidence that the factor structure of the (S)WEMWBS might be best explained by applying a bifactor model (with one general mental well-being factor and three grouping factors) [20, 22]. Results based on other German samples [17, 21, 47] have also shown that the three-factor structure outperforms the one-factor structure for the (S)WEMWBS. The present study achieves an even better fit to the data by applying a bifactor model based on the assumption of one general well-being factor and three subfactors, in line with other evidence [17, 20, 21]. The three factorial structure is theoretically based on the assumption of three well-being dimensions (hedonia, eudaimonia, interpersonal relationships) [12] and has been empirically confirmed in a prior validation study using data from an Austrian German-speaking sample [17]. In consideration of the relatively low common variance of the well-being subdimensions beyond a general mental well-being factor in the SWEMWBS bifactor model, we agree with the conclusion of Lang & Bachinger [17] that total scores can be formed.

Analyses of modification indices showed some improvement of the scales by excluding certain items (e.g., items #7, #11 and #13 for the long version, item #6 for the short version) or the permission of particular cross-loadings as it could be shown in other research as well [20, 22]. However, particular the bifactor models showed good model fit for both, the original short and long versions. These original versions should be given preferences to enable temporal and cultural comparisons and to retain item content of the construct of mental well-being.

Scalar measurement invariance for the both the long (WEMWBS) and short versions (SWEMWBS) was established for sex and age. The results indicate that the items had the same meaning for males and females as well as for younger (< 50 years) and older (> 50 years) adults, justifying comparisons on the mean and intercept level in reporting mental well-being within MHS.

In terms of criterion validity, all associations between the (S)WEMWBS showed in the expected directions. Associations of the short version and the external criteria corresponded with those of the long version. Closer constructs showed strong associations, while correlations to more distant constructs were of medium magnitudes. Correlations between the WHO-5 and the (S)WEMWBS were particularly high. Post-hoc correlations (not reported) showed that the hedonic facets of the (S)WEMWBS were more strongly associated with the WHO-5 than the facets representing eudaimonic and social aspects of well-being, confirming that the WHO-5 captures aspects of hedonic well-being better. Therefore, the use of the (S)WEMWBS can be recommended beyond the use of the WHO-5 and follows the OECD’s call to consider and assess not only hedonic but also eudaimonic aspects of well-being with appropriate instruments [48].

Validity analyses of the scales’ properties further showed that there were no differences in mental well-being by sex. However, we found significant differences between the younger age groups (< 50 years) and the oldest age group, with significantly higher mental well-being in adults above 65 years. These results are in line with those of the Austrian German validation study [17] and with other findings from well-being research using different measurement instruments [49, 50].

The mean values of the (S)WEMWBS were similar to those of other European countries [14]. The score distributions of both the long and short versions were approximately normal with a slight left skew and were thus comparable with those reported in the original validation studies [11, 16]. The obtained distributions indicate that the (S)WEMWBS discriminate sufficiently between different groups and are therefore suitable for use in a German MHS. Internal consistencies show item redundancy in the long version [13] and justify the application of the short version in monitoring population mental well-being. The SWEMWBS also showed similarly high correlations with the external criteria used to estimate criterion validity as the WEMWBS as well as a high correlation with the items of the longer WEMWBS not included in it, further suggesting that the application of the short version is appropriate.

In sum, our results provide evidence in favor of employing the widely-used SWEMWBS in Germany’s MHS with no need to modify the scale. However, it should be noted that neither the long nor the short version entirely covers all three mental well-being dimensions of hedonic, eudaimonic, and social well-being. For instance, *life satisfaction*, representing the cognitive-evaluative aspect of hedonic well-being, is one of the most commonly used indicators of well-being but is not represented in the WEMWBS [51]. In particular, the SWEMWBS mainly comprises items that can be assigned to eudaimonia. Therefore, critical consideration should be given to whether the (S)WEMWBS should be supplemented by additional relevant items for a comprehensive and internationally comparable German MHS (e.g., Satisfaction with Life Scale (SWLS; [52]). Moreover, it should be noted that social well-being as conceptualized by Keyes [8] is not covered by the SWEMWBS. The third factor of the WEMWBS captures social aspects of mental well-being but only with a view to the immediate social environment (i.e., interpersonal relationships), thus not fully covering the broader original concept of eudaimonia, which includes positive ties to society at large [7]. Given the increasing challenges associated with various emerging societal crises [1], well-being indicators capturing individuals’ relationship to broader society may be of growing importance for MHS.

The following limitations should be considered when interpreting the findings of the present study and deriving recommendations for future studies:

First, we used a convenience sample that was not representative of the German population structure (53). Future studies should investigate the properties of the (S)WEMWBS in representative samples, also in order to calculate norm values, which could be used as a benchmark for national and international comparisons both on the population and the individual level. In terms of more evidence-based mental health measures, norms can serve as a reference, for example when conducting impact analyses of targeted interventions for specific subgroups.

Second, data collection fell within the period of the COVID-19 pandemic, which could have impacted participants’ mental well-being at the time of measurement and thus the mean scores of the (S)WEMWBS. Long-term data are needed to replicate the reported findings.

Finally, we did not test the discriminant validity of the scale by examining its association with constructs hypothesized to be uncorrelated to mental well-being and did not conduct qualitative analyses to assess face validity. Future studies in German samples might implement a multimethod approach, for example, for validation among children and youth for whom the comprehensibility of items needs to be demonstrated in particular.

## Conclusions

Positive mental health (i.e., mental well-being) represents an equally important object of comprehensive MHS alongside psychopathology following a dual continua model of mental health. Monitoring mental well-being on a regular basis provides an important evidentiary foundation for prevention and promotion in public mental health, including the development of measures to improve mental well-being tailored to specific subgroups. In fact, higher levels of mental well-being have already been shown to positively influence recovery from affective disorders, showing the potential utility of interventions targeting mental well-being in reducing the burden of mental disorders independent of the degree of psychopathology [54]. To assess mental well-being appropriately, a reliable and internationally comparable measure of well-being is needed for MHS. Conceptual discrepancies and the range of different measurement instruments in existence have posed challenges in identifying a suitable measure. The theoretically derived and empirically sound factorial structure as well as the psychometric properties of the (S)WEMWBS in this German validation study were in line with the original validation studies and previous findings from other countries. The present study therefore provides evidence in support of suitability of the (S)WEMWBS for use in Germany’s MHS for both comprehensive national monitoring of population mental health and international comparisons of mental well-being at the population level. The extent to which the scale should be complemented by further measures of mental well-being to address all dimensions of mental well-being (hedonia, eudaimonia, social) remains to be further investigated and discussed [55, 56].

## Electronic supplementary material

Below is the link to the electronic supplementary material.

Supplementary Material 1

## Data Availability

Since informed consent from study participants did not cover the public deposition of data, the datasets generated and analyzed during the current study are not publicly available but can be provided by the corresponding author on reasonable request.

## Abbreviations

AIC: Akaike Information Criterion
CFA: Confirmatory Factor Analysis
CFI: Comparative Fit Index
MHS: Mental Health Surveillance
OECD: Organization for Economic Cooperation and Development
RKI: Robert Koch Institute
RMSEA: Root Mean Square Error of Approximation
SRMR: Standardized Root mean Square Residual
SWEMWBS: Short Warwick-Edinburgh Mental Well-Being Scale
UK: United Kingdom
WEMWBS: Warwick-Edinburgh Mental Well-Being Scale
WHO: World Health Organization
