# Supplementary material for: Validation of the Warwick-Edinburgh Mental Well-Being Scale for the Mental Health Surveillance (MHS) of German adults
Source: Health Qual Life Outcomes. 2024 Oct 26;22:92. doi: 10.1186/s12955-024-02304-4 (PMC11515111; doi:10.1186/s12955-024-02304-4)
Supplement: Supplementary file 1 — Supplementary Material 1 [file 12955_2024_2304_MOESM1_ESM.docx]

# **Supplementary Materials**

Methods: Description of Questionnaires

In addition to the WEMWBS, the following self-report questionnaires were applied to estimate the criterion validity of the scale’s short and long versions.

### Well-Being

The *WHO-5* measures dimensions of hedonic well-being, such as mood, vitality and interest in activities, with 5 items on a 6-point scale ranging from *not at* all to *all the time.* It is a frequently used questionnaire in larger-scale surveys and has been translated into over 30 languages. The German version showed sound psychometric properties (25-27).

### Health-Related Quality of Life

The *Short-Form Health Survey (SF-12*; (28)) represents the 12-item short version of the SF-36 Health Survey. The screening instrument assesses eight dimensions that allow for the formation of a physical (General Health Perception, Physical Functioning and Role Functioning, and Pain) and psychological (Emotional Role Functioning, Well-Being, Negative Affect, and Social Functioning) health-related quality of life score. Validation studies showed that the SF-12 was as suitable as the longer SF-36 and was preferable in population-based studies (28).

The *Assessment of Quality of Life – 6D* scale (AQoL-6D (29)) was used to measure the impact of a person’s reported health state on life quality and general functioning at the population level with regard to ‘independent living’, ‘relationships’, ‘mental health’, ‘coping’, ‘pain’ and ‘senses’. These six domains of the 20-item self-report questionnaire can be transferred to a physical and psychosocial score and used to form a single global quality of life factor. Response options range from 4 to 6 levels, with higher quality of life impairment shown by higher scores.

The *WHOQoL-BREF* (30) represents the abbreviated version of the WHO quality of life assessment WHOQOL-100. The 26-item self-report measure covers four domains, ‘physical health’, ‘psychological’, ‘social relationships’ and ‘environment’, which can be used to calculate domain scores as well as an overall score of quality of life and general health. Items are answered on a 5-point scale, with higher quality of life impairment shown by lower scores. The short version was closely associated with the long version and suggested for use in larger epidemiological studies (30).

### Psychological Distress

The Kessler-10 distress scale (K-10; (31)) is a common instrument used to assess the frequency of psychological distress (depression and anxiety-related symptoms) over the past four weeks at the population level (31, 32). The 10-item self-report measure is answered on a 5-point scale (1= ‘none of the time’ to 5 =’all of the time’), with higher total scores representing greater psychological distress.

### Psychological Resources

Proactive coping was assessed with the first subscale ‘*proactive coping*’ of the *Proactive Coping Inventory* (PCI: German version). The self-report scale assesses self-regulatory as well goal attainment skills to cope with stressors and to promote overall well-being (34; 35, 55). The 17 items are answered on a 4-point scale (1= ‘not at all true’ to 4 =’completely true’), with higher proactive coping shown by higher scores.

The *Allgemeine Selbstwirksamkeitsskala* (AKSU; (36)) has been applied in a variety of population-based surveys to assess self-efficacy (56). Response options for the 3-item self-report scale range on a 5-point scale from 1= ‘not at all true’ to 5 =’completely true’. The psychometric properties of the AKSU indicate that the scale allows for a reliable and valid assessment of the construct.

**Table S1.1. Results from measurement invariance analyses for configural models of one-factor-solutions separated by sex**

|  | WEMWBS  male | | WEMWBS  female | | SWEMWBS male | | | | | SWEMWBS female | |
| --- | --- | --- | --- | --- | --- | --- | --- | --- | --- | --- | --- |
|  | Estimate | *SE* | Estimate | *SE* | Estimate | | | *SE* | | Estimate | *SE* |
| **Factor loadings** | | | | | | | | | | | |
| WEMWBS 1 | 0.823 | 0.037 | 0.830 | 0.034 | | 0.771 | | 0.040 | | 0.776 | 0.039 |
| WEMWBS 2 | 0.730 | 0.038 | 0.782 | 0.037 | | 0.695 | | 0.040 | | 0.774 | 0.040 |
| WEMWBS 3 | 0.681 | 0.038 | 0.764 | 0.034 | | 0.661 | | 0.039 | | 0.728 | 0.036 |
| WEMWBS 4 | 0.548 | 0.040 | 0.505 | 0.038 | |  | |  | |  |  |
| WEMWBS 5 | 0.775 | 0.033 | 0.811 | 0.031 | |  | |  | |  |  |
| WEMWBS 6 | 0.569 | 0.037 | 0.585 | 0.036 | | 0.623 | | 0.036 | | 0.619 | 0.037 |
| WEMWBS 7 | 0.502 | 0.043 | 0.451 | 0.036 | | 0.576 | | 0.044 | | 0.513 | 0.039 |
| WEMWBS 8 | 0.725 | 0.032 | 0.753 | 0.031 | |  | |  | |  |  |
| WEMWBS 9 | 0.716 | 0.039 | 0.741 | 0.035 | | 0.650 | | 0.042 | | 0.724 | 0.038 |
| WEMWBS 10 | 0.782 | 0.032 | 0.821 | 0.031 | |  | |  | |  |  |
| WEMWBS 11 | 0.474 | 0.041 | 0.502 | 0.036 | | 0.547 | | 0.042 | | 0.559 | 0.037 |
| WEMWBS 12 | 0.724 | 0.041 | 0.755 | 0.041 |  | |  | |  | |  |
| WEMWBS 13 | 0.589 | 0.040 | 0.644 | 0.038 |  | |  | |  | |  |
| WEMWBS 14 | 0.734 | 0.034 | 0.774 | 0.032 |  | |  | |  | |  |
| **Intercepts** |  |  |  |  |  | |  | |  | |  |
| WEMWBS 1 | 3.535 | 0.045 | 3.384 | 0.045 | 3.535 | | 0.045 | | 3.384 | | 0.045 |
| WEMWBS 2 | 3.702 | 0.041 | 3.661 | 0.043 | 3.702 | | 0.041 | | 3.661 | | 0.043 |
| WEMWBS 3 | 3.683 | 0.039 | 3.519 | 0.040 | 3.683 | | 0.039 | | 3.519 | | 0.040 |
| WEMWBS 4 | 3.652 | 0.039 | 3.818 | 0.038 |  | |  | |  | |  |
| WEMWBS 5 | 3.473 | 0.041 | 3.292 | 0.042 |  | |  | |  | |  |
| WEMWBS 6 | 3.919 | 0.036 | 3.943 | 0.035 | 3.919 | | 0.036 | | 3.943 | | 0.035 |
| WEMWBS 7 | 4.260 | 0.036 | 4.316 | 0.033 | 4.260 | | 0.036 | | 4.316 | | 0.033 |
| WEMWBS 8 | 3.769 | 0.037 | 3.701 | 0.038 |  | |  | |  | |  |
| WEMWBS 9 | 3.560 | 0.044 | 3.600 | 0.045 | 3.560 | | 0.044 | | 3.600 | | 0.045 |
| WEMWBS 10 | 3.702 | 0.039 | 3.595 | 0.041 |  | |  | |  | |  |
| WEMWBS 11 | 4.190 | 0.037 | 4.297 | 0.035 | 4.190 | | 0.037 | | 4.297 | | 0.035 |
| WEMWBS 12 | 3.725 | 0.045 | 3.778 | 0.047 |  | |  | |  | |  |
| WEMWBS 13 | 3.637 | 0.041 | 3.581 | 0.043 |  | |  | |  | |  |
| WEMWBS 14 | 3.577 | 0.039 | 3.475 | 0.040 |  | |  | |  | |  |
| **Residual variances** | |  |  |  |  | |  | |  | |  |
| WEMWBS 1 | 0.357 | 0.029 | 0.378 | 0.031 | 0.438 | | 0.038 | | 0.463 | | 0.038 |
| WEMWBS 2 | 0.342 | 0.029 | 0.385 | 0.037 | 0.391 | | 0.036 | | 0.398 | | 0.043 |
| WEMWBS 3 | 0.318 | 0.026 | 0.272 | 0.023 | 0.345 | | 0.031 | | 0.326 | | 0.028 |
| WEMWBS 4 | 0.503 | 0.037 | 0.508 | 0.038 |  | |  | |  | |  |
| WEMWBS 5 | 0.271 | 0.020 | 0.260 | 0.018 |  | |  | |  | |  |
| WEMWBS 6 | 0.335 | 0.037 | 0.291 | 0.021 | 0.271 | | 0.036 | | 0.250 | | 0.022 |
| WEMWBS 7 | 0.406 | 0.029 | 0.388 | 0.026 | 0.326 | | 0.028 | | 0.328 | | 0.029 |
| WEMWBS 8 | 0.198 | 0.019 | 0.188 | 0.016 |  | |  | |  | |  |
| WEMWBS 9 | 0.484 | 0.040 | 0.506 | 0.041 | 0.574 | | 0.051 | | 0.530 | | 0.046 |
| WEMWBS 10 | 0.190 | 0.017 | 0.195 | 0.019 |  | |  | |  | |  |
| WEMWBS 11 | 0.498 | 0.040 | 0.377 | 0.024 | 0.424 | | 0.039 | | 0.317 | | 0.025 |
| WEMWBS 12 | 0.510 | 0.038 | 0.599 | 0.049 |  | |  | |  | |  |
| WEMWBS 13 | 0.516 | 0.045 | 0.567 | 0.041 |  | |  | |  | |  |
| WEMWBS 14 | 0.248 | 0.022 | 0.234 | 0.018 |  | |  | |  | |  |

**Table S1.2. Results from measurement invariance analyses for configural models of three-factor-solutions separated by sex**

|  | WEMWBS  male | | WEMWBS  female | | SWEMWBS  male | | SWEMWBS  female | |
| --- | --- | --- | --- | --- | --- | --- | --- | --- |
|  | Estimate | *SE* | Estimate | *SE* | Estimate | *SE* | Estimate | *SE* |
| **Factor loadings** | |  |  |  |  |  |  |  |
| Eudaimonic ~ | |  |  |  |  |  |  |  |
| WEMWBS 6 | 0.638 | 0.035 | 0.655 | 0.036 | 0.638 | 0.040 | 0.644 | 0.040 |
| WEMWBS 7 | 0.618 | 0.040 | 0.552 | 0.038 | 0.647 | 0.041 | 0.576 | 0.039 |
| WEMWBS 11 | 0.604 | 0.042 | 0.589 | 0.036 | 0.626 | 0.042 | 0.621 | 0.036 |
| WEMWBS 13 | 0.574 | 0.043 | 0.655 | 0.041 |  |  |  |  |
| Hedonic ~ | |  |  |  |  |  |  |  |
| WEMWBS 1 | 0.829 | 0.037 | 0.837 | 0.034 | 0.817 | 0.041 | 0.820 | 0.038 |
| WEMWBS 3 | 0.689 | 0.038 | 0.770 | 0.034 | 0.678 | 0.040 | 0.747 | 0.037 |
| WEMWBS 5 | 0.779 | 0.033 | 0.820 | 0.031 |  |  |  |  |
| WEMWBS 8 | 0.732 | 0.033 | 0.758 | 0.031 |  |  |  |  |
| WEMWBS 10 | 0.786 | 0.033 | 0.827 | 0.031 |  |  |  |  |
| WEMWBS 14 | 0.741 | 0.034 | 0.787 | 0.031 |  |  |  |  |
| Interpersonal  Relationships ~ | |  |  |  |  |  |  |  |
| WEMWBS 2 | 0.742 | 0.040 | 0.816 | 0.038 | 0.730 | 0.044 | 0.844 | 0.041 |
| WEMWBS 4 | 0.589 | 0.041 | 0.544 | 0.039 |  |  |  |  |
| WEMWBS 9 | 0.783 | 0.040 | 0.826 | 0.036 | 0.714 | 0.043 | 0.784 | 0.036 |
| WEMWBS 12 | 0.789 | 0.041 | 0.813 | 0.043 |  |  |  |  |
| **Intercepts** |  |  |  |  |  |  |  |  |
| WEMWBS 6 | 3.919 | 0.036 | 3.943 | 0.035 | 3.919 | 0.036 | 3.943 | 0.035 |
| WEMWBS 7 | 4.260 | 0.036 | 4.316 | 0.033 | 4.260 | 0.036 | 4.316 | 0.033 |
| WEMWBS 11 | 4.190 | 0.037 | 4.297 | 0.035 | 4.190 | 0.037 | 4.297 | 0.035 |
| WEMWBS 13 | 3.637 | 0.041 | 3.581 | 0.043 |  |  |  |  |
| WEMWBS 1 | 3.535 | 0.045 | 3.384 | 0.045 | 3.535 | 0.045 | 3.384 | 0.045 |
| WEMWBS 3 | 3.683 | 0.039 | 3.519 | 0.040 | 3.683 | 0.039 | 3.519 | 0.040 |
| WEMWBS 5 | 3.473 | 0.041 | 3.292 | 0.042 |  |  |  |  |
| WEMWBS 8 | 3.769 | 0.037 | 3.701 | 0.038 |  |  |  |  |
| WEMWBS 10 | 3.702 | 0.039 | 3.595 | 0.041 |  |  |  |  |
| WEMWBS 14 | 3.577 | 0.039 | 3.475 | 0.040 |  |  |  |  |
| WEMWBS 2 | 3.702 | 0.041 | 3.661 | 0.043 | 3.702 | 0.041 | 3.661 | 0.043 |
| WEMWBS 4 | 3.652 | 0.039 | 3.818 | 0.038 |  |  |  |  |
| WEMWBS 9 | 3.560 | 0.044 | 3.600 | 0.045 | 3.560 | 0.044 | 3.600 | 0.045 |
| WEMWBS 12 | 3.725 | 0.045 | 3.778 | 0.047 |  |  |  |  |
| **Covariances** |  |  |  |  |  |  |  |  |
| Eudaimonic ~ | |  |  |  |  |  |  |  |
| Hedonic | 0.827 | 0.031 | 0.836 | 0.024 | 0.812 | 0.048 | 0.833 | 0.031 |
| Interpersonal Relationships | 0.775 | 0.041 | 0.823 | 0.031 | 0.775 | 0.052 | 0.775 | 0.037 |
| Hedonic ~ |  |  |  |  |  |  |  |  |
| Interpersonal Relationships | 0.910 | 0.019 | 0.892 | 0.018 | 0.949 | 0.037 | 0.907 | 0.028 |
| **Residual variances** | |  |  |  |  |  |  |  |
| WEMWBS 6 | 0.252 | 0.036 | 0.204 | 0.022 | 0.252 | 0.042 | 0.218 | 0.026 |
| WEMWBS 7 | 0.275 | 0.026 | 0.287 | 0.030 | 0.239 | 0.029 | 0.259 | 0.033 |
| WEMWBS 11 | 0.359 | 0.040 | 0.282 | 0.027 | 0.331 | 0.042 | 0.244 | 0.028 |
| WEMWBS 13 | 0.532 | 0.052 | 0.553 | 0.042 |  |  |  |  |
| WEMWBS 1 | 0.346 | 0.031 | 0.365 | 0.031 | 0.366 | 0.042 | 0.393 | 0.039 |
| WEMWBS 3 | 0.307 | 0.027 | 0.262 | 0.024 | 0.322 | 0.031 | 0.297 | 0.031 |
| WEMWBS 5 | 0.265 | 0.021 | 0.246 | 0.018 |  |  |  |  |
| WEMWBS 8 | 0.188 | 0.018 | 0.180 | 0.016 |  |  |  |  |
| WEMWBS 10 | 0.183 | 0.018 | 0.186 | 0.020 |  |  |  |  |
| WEMWBS 14 | 0.238 | 0.021 | 0.214 | 0.017 |  |  |  |  |
| WEMWBS 2 | 0.324 | 0.032 | 0.331 | 0.042 | 0.342 | 0.044 | 0.284 | 0.049 |
| WEMWBS 4 | 0.457 | 0.040 | 0.467 | 0.036 |  |  |  |  |
| WEMWBS 9 | 0.384 | 0.038 | 0.373 | 0.038 | 0.487 | 0.049 | 0.440 | 0.042 |
| WEMWBS 12 | 0.411 | 0.037 | 0.508 | 0.049 |  |  |  |  |

**Table S1.3. Results from measurement invariance analyses for the configural models of bifactor-factor-solutions separated by sex**

|  | WEMWBS  male | | WEMWBS  female | | SWEMWBS  male | | SWEMWBS  female | |
| --- | --- | --- | --- | --- | --- | --- | --- | --- |
|  | Estimate | *SE* | Estimate | *SE* | Estimate | *SE* | Estimate | *SE* |
| **Factor loadings** | |  |  |  |  |  |  |  |
| Well-being ~ |  |  |  |  |  |  |  |  |
| WEMWBS 1 | 1.253 | 0.109 | 1.140 | 0.188 | 1.016 | 0.085 | 1.023 | 0.096 |
| WEMWBS 2 | 1.135 | 0.079 | 1.132 | 0.138 | 0.972 | 0.076 | 1.043 | 0.084 |
| WEMWBS 3 | 1.076 | 0.086 | 1.126 | 0.161 | 0.917 | 0.064 | 1.052 | 0.076 |
| WEMWBS 4 | 0.832 | 0.068 | 0.783 | 0.066 |  |  |  |  |
| WEMWBS 5 | 1.175 | 0.092 | 1.126 | 0.169 |  |  |  |  |
| WEMWBS 6 | 1.000 |  | 1.000 |  | 1.000 |  | 1.000 |  |
| WEMWBS 7 | 0.991 | 0.070 | 0.862 | 0.076 | 0.883 | 0.066 | 0.821 | 0.077 |
| WEMWBS 8 | 1.130 | 0.083 | 1.095 | 0.153 |  |  |  |  |
| WEMWBS 9 | 0.985 | 0.088 | 1.003 | 0.105 | 0.818 | 0.088 | 0.958 | 0.082 |
| WEMWBS 10 | 1.196 | 0.094 | 1.175 | 0.165 |  |  |  |  |
| WEMWBS 11 | 0.992 | 0.078 | 0.905 | 0.059 | 0.854 | 0.078 | 0.868 | 0.069 |
| WEMWBS 12 | 1.048 | 0.092 | 1.036 | 0.183 |  |  |  |  |
| WEMWBS 13 | 0.943 | 0.069 | 1.042 | 0.065 |  |  |  |  |
| WEMWBS 14 | 1.091 | 0.103 | 1.022 | 0.193 |  |  |  |  |
| Eudaimonic ~ | |  |  |  |  |  |  |  |
| WEMWBS 6 |  |  |  |  |  |  |  |  |
| WEMWBS 7 | 1.000 |  | 1.000 |  | 0.285 | 0.578 | 6.558 | 31.466 |
| WEMWBS 11 | 1.403 | 0.559 | 0.694 | 0.599 | 1.000 |  | 1.000 |  |
| WEMWBS 13 | -0.471 | 0.419 | -0.395 | 0.831 |  |  |  |  |
| Hedonic ~ | |  |  |  |  |  |  |  |
| WEMWBS 1 | 1.000 |  | 1.000 |  | 1.000 |  | 1.000 |  |
| WEMWBS 3 | 0.644 | 0.216 | 0.651 | 0.206 | 0.500 | 0.160 | 0.331 | 0.122 |
| WEMWBS 5 | 0.977 | 0.194 | 0.972 | 0.162 |  |  |  |  |
| WEMWBS 8 | 0.746 | 0.167 | 0.698 | 0.156 |  |  |  |  |
| WEMWBS 10 | 0.911 | 0.139 | 0.819 | 0.093 |  |  |  |  |
| WEMWBS 14 | 1.074 | 0.186 | 1.158 | 0.182 |  |  |  |  |
| Interpersonal  Relationships ~ | |  |  |  |  |  |  |  |
| WEMWBS 2 | 1.000 |  | 1.000 |  | 1.000 |  | 1.000 |  |
| WEMWBS 4 | 1.463 | 0.636 | 0.697 | 0.706 |  |  |  |  |
| WEMWBS 9 | 3.112 | 1.510 | 1.893 | 1.382 | 2.079 | 0.652 | 0.906 | 0.177 |
| WEMWBS 12 | 2.361 | 0.759 | 1.502 | 0.292 |  |  |  |  |
| **Intercepts** |  |  |  |  |  |  |  |  |
| WEMWBS 6 | 0.000 |  | 0.000 |  | 0.000 |  | 0.000 |  |
| WEMWBS 7 | 0.000 |  | 0.000 |  | 0.557 | 0.613 | -4.671 | 28.507 |
| WEMWBS 11 | -0.224 | 0.581 | 0.093 | 0.729 | 0.000 |  | 0.000 |  |
| WEMWBS 13 | 0.118 | 0.347 | -0.166 | 0.876 |  |  |  |  |
| WEMWBS 1 | 0.000 |  | 0.000 |  | 0.000 |  | 0.000 |  |
| WEMWBS 3 | 0.352 | 0.419 | -0.199 | 0.431 | 0.313 | 0.249 | -0.413 | 0.262 |
| WEMWBS 5 | 0.211 | 0.380 | -0.068 | 0.352 |  |  |  |  |
| WEMWBS 8 | 0.368 | 0.301 | 0.156 | 0.331 |  |  |  |  |
| WEMWBS 10 | 0.269 | 0.319 | -0.130 | 0.223 |  |  |  |  |
| WEMWBS 14 | 0.778 | 0.375 | 0.732 | 0.384 |  |  |  |  |
| WEMWBS 2 | 0.000 |  | 0.000 |  | 0.000 |  | 0.000 |  |
| WEMWBS 4 | 1.483 | 0.744 | 1.290 | 1.012 |  |  |  |  |
| WEMWBS 9 | 2.027 | 1.671 | 1.167 | 1.741 | 0.575 | 0.527 | 0.235 | 0.281 |
| WEMWBS 12 | 1.383 | 0.958 | 0.901 | 0.473 |  |  |  |  |
| **Covariances** |  |  |  |  |  |  |  |  |
| Well-being ~~ |  |  |  |  |  |  |  |  |
| Eudaimonic | 0.000 |  | 0.000 |  | 0.000 |  | 0.000 |  |
| Hedonic | 0.000 |  | 0.000 |  | 0.000 |  | 0.000 |  |
| Interpersonal relationships | 0.000 |  | 0.000 |  | 0.000 |  | 0.000 |  |
| Eudaimonic ~ | |  |  |  |  |  |  |  |
| Hedonic | -0.074 | 0.031 | -0.069 | 0.061 | -0.022 | 0.041 | -0.007 | 0.035 |
| Interpersonal Relationships | -0.023 | 0.016 | -0.020 | 0.018 | -0.024 | 0.018 | -0.004 | 0.018 |
| Hedonic ~ |  |  |  |  |  |  |  |  |
| Interpersonal Relationships | 0.038 | 0.031 | 0.073 | 0.100 | 0.112 | 0.048 | 0.198 | 0.046 |
| **Residual variances** | |  |  |  |  |  |  |  |
| WEMWBS 6 | 0.285 | 0.036 | 0.223 | 0.042 | 0.214 | 0.038 | 0.196 | 0.029 |
| WEMWBS 7 | 0.231 | 0.047 | 0.172 | 0.122 | 0.278 | 0.080 | -0.273 | 2.807 |
| WEMWBS 11 | 0.240 | 0.074 | 0.239 | 0.079 | -0.005 | 0.841 | 0.287 | 0.083 |
| WEMWBS 13 | 0.516 | 0.049 | 0.519 | 0.100 |  |  |  |  |
| WEMWBS 1 | 0.347 | 0.032 | 0.365 | 0.034 | 0.295 | 0.087 | 0.179 | 0.143 |
| WEMWBS 3 | 0.308 | 0.026 | 0.264 | 0.023 | 0.338 | 0.030 | 0.325 | 0.029 |
| WEMWBS 5 | 0.261 | 0.022 | 0.240 | 0.020 |  |  |  |  |
| WEMWBS 8 | 0.191 | 0.018 | 0.181 | 0.015 |  |  |  |  |
| WEMWBS 10 | 0.185 | 0.017 | 0.191 | 0.020 |  |  |  |  |
| WEMWBS 14 | 0.227 | 0.022 | 0.179 | 0.021 |  |  |  |  |
| WEMWBS 2 | 0.351 | 0.029 | 0.364 | 0.050 | 0.374 | 0.037 | 0.272 | 0.054 |
| WEMWBS 4 | 0.455 | 0.041 | 0.459 | 0.037 |  |  |  |  |
| WEMWBS 9 | 0.229 | 0.084 | 0.258 | 0.211 | 0.351 | 0.090 | 0.450 | 0.050 |
| WEMWBS 12 | 0.391 | 0.044 | 0.487 | 0.102 |  |  |  |  |

**Table S1.4. Results from measurement invariance analyses for the most constrained models (loadings, intercepts) of one-factor solutions separated by sex**

|  | WEMWBS  male | | | | WEMWBS  female | | SWEMWBS male | | | SWEMWBS female | |
| --- | --- | --- | --- | --- | --- | --- | --- | --- | --- | --- | --- |
|  | Estimate | | *SE* | | Estimate | *SE* | Estimate | *SE* | | Estimate | *SE* |
| **Factor loadings** | |  | |  |  |  |  |  |  |  |  |
| WEMWBS 1 | 0.810 | | 0.033 | | 0.810 | 0.033 | 0.761 | 0.035 | | 0.761 | 0.035 |
| WEMWBS 2 | 0.739 | | 0.033 | | 0.739 | 0.033 | 0.722 | 0.034 | | 0.722 | 0.034 |
| WEMWBS 3 | 0.711 | | 0.030 | | 0.711 | 0.030 | 0.684 | 0.033 | | 0.684 | 0.033 |
| WEMWBS 4 | 0.509 | | 0.032 | | 0.509 | 0.032 |  |  | |  |  |
| WEMWBS 5 | 0.778 | | 0.030 | | 0.778 | 0.030 |  |  | |  |  |
| WEMWBS 6 | 0.562 | | 0.030 | | 0.562 | 0.030 | 0.608 | 0.033 | | 0.608 | 0.033 |
| WEMWBS 7 | 0.461 | | 0.032 | | 0.461 | 0.032 | 0.531 | 0.036 | | 0.531 | 0.036 |
| WEMWBS 8 | 0.723 | | 0.029 | | 0.723 | 0.029 |  |  | |  |  |
| WEMWBS 9 | 0.710 | | 0.033 | | 0.710 | 0.033 | 0.676 | 0.035 | | 0.676 | 0.035 |
| WEMWBS 10 | 0.785 | | 0.030 | | 0.785 | 0.030 |  |  | |  |  |
| WEMWBS 11 | 0.474 | | 0.029 | | 0.474 | 0.029 | 0.541 | 0.032 | | 0.541 | 0.032 |
| WEMWBS 12 | 0.721 | | 0.035 | | 0.721 | 0.035 |  |  |  | |  |
| WEMWBS 13 | 0.603 | | 0.031 | | 0.603 | 0.031 |  |  |  | |  |
| WEMWBS 14 | 0.739 | | 0.030 | | 0.739 | 0.030 |  |  |  | |  |
| **Intercepts** |  | |  | |  |  |  |  |  | |  |
| WEMWBS 1 | 3.497 | | 0.041 | | 3.497 | 0.041 | 3.476 | 0.041 | 3.476 | | 0.041 |
| WEMWBS 2 | 3.715 | | 0.037 | | 3.715 | 0.037 | 3.696 | 0.038 | 3.696 | | 0.038 |
| WEMWBS 3 | 3.629 | | 0.036 | | 3.629 | 0.036 | 3.613 | 0.036 | 3.613 | | 0.036 |
| WEMWBS 4 | 3.759 | | 0.033 | | 3.759 | 0.033 |  |  |  | |  |
| WEMWBS 5 | 3.417 | | 0.039 | | 3.417 | 0.039 |  |  |  | |  |
| WEMWBS 6 | 3.960 | | 0.030 | | 3.960 | 0.030 | 3.945 | 0.032 | 3.945 | | 0.032 |
| WEMWBS 7 | 4.311 | | 0.028 | | 4.311 | 0.028 | 4.300 | 0.030 | 4.300 | | 0.030 |
| WEMWBS 8 | 3.768 | | 0.035 | | 3.768 | 0.035 |  |  |  | |  |
| WEMWBS 9 | 3.612 | | 0.038 | | 3.612 | 0.038 | 3.594 | 0.038 | 3.594 | | 0.038 |
| WEMWBS 10 | 3.684 | | 0.037 | | 3.684 | 0.037 |  |  |  | |  |
| WEMWBS 11 | 4.277 | | 0.028 | | 4.277 | 0.028 | 4.265 | 0.030 | 4.265 | | 0.030 |
| WEMWBS 12 | 3.780 | | 0.039 | | 3.780 | 0.039 |  |  |  | |  |
| WEMWBS 13 | 3.637 | | 0.035 | | 3.637 | 0.035 |  |  |  | |  |
| WEMWBS 14 | 3.559 | | 0.036 | | 3.559 | 0.036 |  |  |  | |  |
| **Residual variances** | | |  | |  |  |  |  |  | |  |
| WEMWBS 1 | 0.359 | | 0.029 | | 0.379 | 0.031 | 0.438 | 0.036 | 0.472 | | 0.038 |
| WEMWBS 2 | 0.342 | | 0.029 | | 0.385 | 0.036 | 0.382 | 0.035 | 0.406 | | 0.041 |
| WEMWBS 3 | 0.320 | | 0.026 | | 0.277 | 0.024 | 0.346 | 0.031 | 0.335 | | 0.029 |
| WEMWBS 4 | 0.519 | | 0.038 | | 0.519 | 0.038 |  |  |  | |  |
| WEMWBS 5 | 0.274 | | 0.020 | | 0.264 | 0.019 |  |  |  | |  |
| WEMWBS 6 | 0.338 | | 0.036 | | 0.292 | 0.021 | 0.277 | 0.035 | 0.247 | | 0.022 |
| WEMWBS 7 | 0.412 | | 0.029 | | 0.390 | 0.026 | 0.340 | 0.027 | 0.324 | | 0.028 |
| WEMWBS 8 | 0.198 | | 0.019 | | 0.188 | 0.016 |  |  |  | |  |
| WEMWBS 9 | 0.489 | | 0.041 | | 0.509 | 0.040 | 0.565 | 0.050 | 0.539 | | 0.045 |
| WEMWBS 10 | 0.190 | | 0.017 | | 0.195 | 0.019 |  |  |  | |  |
| WEMWBS 11 | 0.508 | | 0.041 | | 0.381 | 0.025 | 0.437 | 0.040 | 0.316 | | 0.025 |
| WEMWBS 12 | 0.514 | | 0.038 | | 0.603 | 0.049 |  |  |  | |  |
| WEMWBS 13 | 0.516 | | 0.045 | | 0.567 | 0.040 |  |  |  | |  |
| WEMWBS 14 | 0.247 | | 0.021 | | 0.235 | 0.018 |  |  |  | |  |

**Table S.1.5. Results from measurement invariance analyses for the most constrained models (loadings, intercepts) of three-factor-solutions separated by sex**

|  | WEMWBS  male | | WEMWBS  female | | SWEMWBS  male | | SWEMWBS  female | |
| --- | --- | --- | --- | --- | --- | --- | --- | --- |
|  | Estimate | *SE* | Estimate | *SE* | Estimate | *SE* | Estimate | *SE* |
| **Factor loadings** | |  |  |  |  |  |  |  |
| Eudaimonic ~ | |  |  |  |  |  |  |  |
| WEMWBS 6 | 0.647 | 0.032 | 0.647 | 0.032 | 0.652 | 0.034 | 0.652 | 0.034 |
| WEMWBS 7 | 0.583 | 0.037 | 0.583 | 0.037 | 0.621 | 0.038 | 0.621 | 0.038 |
| WEMWBS 11 | 0.596 | 0.035 | 0.596 | 0.035 | 0.637 | 0.035 | 0.637 | 0.035 |
| WEMWBS 13 | 0.612 | 0.033 | 0.612 | 0.033 |  |  |  |  |
| Hedonic ~ | |  |  |  |  |  |  |  |
| WEMWBS 1 | 0.812 | 0.033 | 0.812 | 0.033 | 0.797 | 0.040 | 0.797 | 0.040 |
| WEMWBS 3 | 0.715 | 0.030 | 0.715 | 0.030 | 0.697 | 0.036 | 0.697 | 0.036 |
| WEMWBS 5 | 0.781 | 0.030 | 0.781 | 0.030 |  |  |  |  |
| WEMWBS 8 | 0.723 | 0.029 | 0.723 | 0.029 |  |  |  |  |
| WEMWBS 10 | 0.785 | 0.030 | 0.785 | 0.030 |  |  |  |  |
| WEMWBS 14 | 0.744 | 0.030 | 0.744 | 0.030 |  |  |  |  |
| Interpersonal  Relationships ~ | |  |  |  |  |  |  |  |
| WEMWBS 2 | 0.759 | 0.035 | 0.759 | 0.035 | 0.740 | 0.040 | 0.740 | 0.040 |
| WEMWBS 4 | 0.553 | 0.034 | 0.553 | 0.034 |  |  |  |  |
| WEMWBS 9 | 0.785 | 0.036 | 0.785 | 0.036 | 0.700 | 0.040 | 0.700 | 0.040 |
| WEMWBS 12 | 0.783 | 0.038 | 0.783 | 0.038 |  |  |  |  |
| **Intercepts** |  |  |  |  |  |  |  |  |
| WEMWBS 6 | 3.909 | 0.033 | 3.909 | 0.033 | 3.900 | 0.034 | 3.900 | 0.034 |
| WEMWBS 7 | 4.269 | 0.032 | 4.269 | 0.032 | 4.259 | 0.033 | 4.259 | 0.033 |
| WEMWBS 11 | 4.229 | 0.033 | 4.229 | 0.033 | 4.218 | 0.034 | 4.218 | 0.034 |
| WEMWBS 13 | 3.590 | 0.036 | 3.590 | 0.036 |  |  |  |  |
| WEMWBS 1 | 3.525 | 0.041 | 3.525 | 0.041 | 3.544 | 0.042 | 3.544 | 0.042 |
| WEMWBS 3 | 3.657 | 0.036 | 3.657 | 0.036 | 3.674 | 0.037 | 3.674 | 0.037 |
| WEMWBS 5 | 3.444 | 0.038 | 3.444 | 0.038 |  |  |  |  |
| WEMWBS 8 | 3.794 | 0.035 | 3.794 | 0.035 |  |  |  |  |
| WEMWBS 10 | 3.712 | 0.037 | 3.712 | 0.037 |  |  |  |  |
| WEMWBS 14 | 3.587 | 0.036 | 3.587 | 0.036 |  |  |  |  |
| WEMWBS 2 | 3.663 | 0.039 | 3.663 | 0.039 | 3.685 | 0.040 | 3.685 | 0.040 |
| WEMWBS 4 | 3.721 | 0.034 | 3.721 | 0.034 |  |  |  |  |
| WEMWBS 9 | 3.560 | 0.041 | 3.560 | 0.041 | 3.586 | 0.040 | 3.586 | 0.040 |
| WEMWBS 12 | 3.731 | 0.042 | 3.731 | 0.042 |  |  |  |  |
| **Covariances** |  |  |  |  |  |  |  |  |
| Eudaimonic ~ | |  |  |  |  |  |  |  |
| Hedonic | 0.836 | 0.029 | 0.875 | 0.097 | 0.818 | 0.045 | 0.840 | 0.099 |
| Interpersonal Relationships | 0.784 | 0.038 | 0.858 | 0.098 | 0.781 | 0.049 | 0.846 | 0.104 |
| Hedonic ~ |  |  |  |  |  |  |  |  |
| Interpersonal Relationships | 0.912 | 0.018 | 0.983 | 0.097 | 0.946 | 0.037 | 1.083 | 0.120 |
| **Residual variances** | |  |  |  |  |  |  |  |
| WEMWBS 6 | 0.250 | 0.035 | 0.207 | 0.021 | 0.245 | 0.038 | 0.225 | 0.024 |
| WEMWBS 7 | 0.288 | 0.026 | 0.280 | 0.029 | 0.249 | 0.026 | 0.252 | 0.031 |
| WEMWBS 11 | 0.368 | 0.039 | 0.277 | 0.026 | 0.334 | 0.040 | 0.243 | 0.027 |
| WEMWBS 13 | 0.525 | 0.049 | 0.567 | 0.042 |  |  |  |  |
| WEMWBS 1 | 0.349 | 0.030 | 0.363 | 0.031 | 0.375 | 0.041 | 0.386 | 0.038 |
| WEMWBS 3 | 0.307 | 0.027 | 0.265 | 0.023 | 0.316 | 0.030 | 0.304 | 0.030 |
| WEMWBS 5 | 0.266 | 0.021 | 0.248 | 0.019 |  |  |  |  |
| WEMWBS 8 | 0.190 | 0.018 | 0.181 | 0.016 |  |  |  |  |
| WEMWBS 10 | 0.183 | 0.017 | 0.185 | 0.019 |  |  |  |  |
| WEMWBS 14 | 0.237 | 0.021 | 0.214 | 0.017 |  |  |  |  |
| WEMWBS 2 | 0.322 | 0.030 | 0.340 | 0.041 | 0.337 | 0.043 | 0.288 | 0.046 |
| WEMWBS 4 | 0.468 | 0.040 | 0.469 | 0.036 |  |  |  |  |
| WEMWBS 9 | 0.387 | 0.037 | 0.371 | 0.037 | 0.495 | 0.049 | 0.437 | 0.041 |
| WEMWBS 12 | 0.414 | 0.036 | 0.506 | 0.048 |  |  |  |  |

**Table S1.6. Results from measurement invariance analyses for the most constrained models (loadings, intercepts) of bifactor-solutions separated by sex**

|  | WEMWBS  male | | WEMWBS  female | | SWEMWBS  male | | SWEMWBS  female | |
| --- | --- | --- | --- | --- | --- | --- | --- | --- |
|  | Estimate | *SE* | Estimate | *SE* | Estimate | *SE* | Estimate | *SE* |
| **Factor loadings** | |  |  |  |  |  |  |  |
| Well-being ~ |  |  |  |  |  |  |  |  |
| WEMWBS 1 | 1.185 | 0.081 | 1.185 | 0.081 | 0.963 | 0.067 | 0.963 | 0.067 |
| WEMWBS 2 | 1.134 | 0.063 | 1.134 | 0.063 | 0.953 | 0.065 | 0.953 | 0.065 |
| WEMWBS 3 | 1.091 | 0.071 | 1.091 | 0.071 | 0.938 | 0.053 | 0.938 | 0.053 |
| WEMWBS 4 | 0.806 | 0.046 | 0.806 | 0.046 |  |  |  |  |
| WEMWBS 5 | 1.138 | 0.071 | 1.138 | 0.071 |  |  |  |  |
| WEMWBS 6 | 1.000 |  | 1.000 |  | 1.000 |  | 1.000 |  |
| WEMWBS 7 | 0.910 | 0.050 | 0.910 | 0.050 | 0.812 | 0.052 | 0.812 | 0.052 |
| WEMWBS 8 | 1.101 | 0.066 | 1.101 | 0.066 |  |  |  |  |
| WEMWBS 9 | 0.990 | 0.063 | 0.990 | 0.063 | 0.833 | 0.059 | 0.833 | 0.059 |
| WEMWBS 10 | 1.175 | 0.073 | 1.175 | 0.073 |  |  |  |  |
| WEMWBS 11 | 0.949 | 0.047 | 0.949 | 0.047 | 0.821 | 0.059 | 0.821 | 0.059 |
| WEMWBS 12 | 1.035 | 0.077 | 1.035 | 0.077 |  |  |  |  |
| WEMWBS 13 | 0.991 | 0.048 | 0.991 | 0.048 |  |  |  |  |
| WEMWBS 14 | 1.046 | 0.083 | 1.046 | 0.083 |  |  |  |  |
| Eudaimonic ~ | |  |  |  |  |  |  |  |
| WEMWBS 6 |  |  |  |  |  |  |  |  |
| WEMWBS 7 | 1.000 |  | 1.000 |  | 0.474 | 0.475 | 0.474 | 0.475 |
| WEMWBS 11 | 1.304 | 0.597 | 1.304 | 0.597 | 1.000 |  | 1.000 |  |
| WEMWBS 13 | -0.613 | 0.424 | -0.613 | 0.424 |  |  |  |  |
| Hedonic ~ | |  |  |  |  |  |  |  |
| WEMWBS 1 | 1.000 |  | 1.000 |  | 1.000 |  | 1.000 |  |
| WEMWBS 3 | 0.694 | 0.127 | 0.694 | 0.127 | 0.524 | 0.096 | 0.524 | 0.096 |
| WEMWBS 5 | 0.988 | 0.119 | 0.988 | 0.119 |  |  |  |  |
| WEMWBS 8 | 0.717 | 0.101 | 0.717 | 0.101 |  |  |  |  |
| WEMWBS 10 | 0.853 | 0.068 | 0.853 | 0.068 |  |  |  |  |
| WEMWBS 14 | 1.089 | 0.109 | 1.089 | 0.109 |  |  |  |  |
| Interpersonal  Relationships ~ | |  |  |  |  |  |  |  |
| WEMWBS 2 | 1.000 |  | 1.000 |  | 1.000 |  | 1.000 |  |
| WEMWBS 4 | 1.035 | 0.374 | 1.035 | 0.374 |  |  |  |  |
| WEMWBS 9 | 2.429 | 0.764 | 2.429 | 0.764 | 1.282 | 0.226 | 1.282 | 0.226 |
| WEMWBS 12 | 1.893 | 0.314 | 1.893 | 0.314 |  |  |  |  |
| **Intercepts** |  |  |  |  |  |  |  |  |
| WEMWBS 6 | 0.000 |  | 0.000 |  | 0.000 |  | 0.000 |  |
| WEMWBS 7 | 0.000 |  | 0.000 |  | 0.613 | 0.507 | 0.613 | 0.507 |
| WEMWBS 11 | -0.414 | 0.628 | -0.414 | 0.628 | 0.000 |  | 0.000 |  |
| WEMWBS 13 | 0.146 | 0.385 | 0.146 | 0.385 |  |  |  |  |
| WEMWBS 1 | 0.000 |  | 0.000 |  | 0.000 |  | 0.000 |  |
| WEMWBS 3 | 0.138 | 0.267 | 0.138 | 0.267 | 0.083 | 0.165 | 0.083 | 0.165 |
| WEMWBS 5 | 0.091 | 0.251 | 0.091 | 0.251 |  |  |  |  |
| WEMWBS 8 | 0.264 | 0.206 | 0.264 | 0.206 |  |  |  |  |
| WEMWBS 10 | 0.052 | 0.172 | 0.052 | 0.172 |  |  |  |  |
| WEMWBS 14 | 0.721 | 0.234 | 0.721 | 0.234 |  |  |  |  |
| WEMWBS 2 | 0.000 |  | 0.000 |  | 0.000 |  | 0.000 |  |
| WEMWBS 4 | 1.372 | 0.494 | 1.372 | 0.494 |  |  |  |  |
| WEMWBS 9 | 1.572 | 0.916 | 1.572 | 0.916 | 0.394 | 0.248 | 0.394 | 0.248 |
| WEMWBS 12 | 1.151 | 0.460 | 1.151 | 0.460 |  |  |  |  |
| **Covariances** |  |  |  |  |  |  |  |  |
| Well-being ~~ |  |  |  |  |  |  |  |  |
| Eudaimonic | 0.000 |  | 0.000 |  | 0.000 |  | 0.000 |  |
| Hedonic | 0.000 |  | 0.000 |  | 0.000 |  | 0.000 |  |
| Interpersonal relationships | 0.000 |  | 0.000 |  | 0.000 |  | 0.000 |  |
| Eudaimonic ~ | |  |  |  |  |  |  |  |
| Hedonic | -0.069 | 0.026 | -0.047 | 0.038 | 0.000 | 0.049 | 0.013 | 0.046 |
| Interpersonal Relationships | -0.029 | 0.012 | -0.012 | 0.011 | -0.017 | 0.031 | 0.021 | 0.030 |
| Hedonic ~ |  |  |  |  |  |  |  |  |
| Interpersonal Relationships | 0.056 | 0.030 | 0.054 | 0.036 | 0.164 | 0.041 | 0.194 | 0.057 |
| **Residual variances** | |  |  |  |  |  |  |  |
| WEMWBS 6 | 0.277 | 0.035 | 0.225 | 0.026 | 0.199 | 0.037 | 0.166 | 0.032 |
| WEMWBS 7 | 0.232 | 0.046 | 0.234 | 0.049 | 0.262 | 0.065 | 0.265 | 0.054 |
| WEMWBS 11 | 0.246 | 0.068 | 0.179 | 0.069 | 0.143 | 0.276 | 0.099 | 0.222 |
| WEMWBS 13 | 0.502 | 0.048 | 0.517 | 0.065 |  |  |  |  |
| WEMWBS 1 | 0.349 | 0.031 | 0.363 | 0.033 | 0.299 | 0.072 | 0.298 | 0.064 |
| WEMWBS 3 | 0.308 | 0.026 | 0.268 | 0.023 | 0.337 | 0.030 | 0.321 | 0.028 |
| WEMWBS 5 | 0.261 | 0.022 | 0.239 | 0.020 |  |  |  |  |
| WEMWBS 8 | 0.192 | 0.018 | 0.182 | 0.015 |  |  |  |  |
| WEMWBS 10 | 0.187 | 0.017 | 0.189 | 0.020 |  |  |  |  |
| WEMWBS 14 | 0.224 | 0.022 | 0.190 | 0.020 |  |  |  |  |
| WEMWBS 2 | 0.345 | 0.028 | 0.375 | 0.040 | 0.355 | 0.039 | 0.328 | 0.047 |
| WEMWBS 4 | 0.466 | 0.040 | 0.457 | 0.034 |  |  |  |  |
| WEMWBS 9 | 0.251 | 0.073 | 0.234 | 0.076 | 0.447 | 0.056 | 0.387 | 0.058 |
| WEMWBS 12 | 0.386 | 0.042 | 0.491 | 0.058 |  |  |  |  |

**Table S2.1. Results from measurement invariance analyses for configural models of one-factor-solutions separated by age**

|  | WEMWBS  < 50 y. | | | | WEMWBS  >= 50 y. | | SWEMWBS  < 50 y. | | | SWEMWBS  >= 50 y. | |
| --- | --- | --- | --- | --- | --- | --- | --- | --- | --- | --- | --- |
|  | Estimate | | *SE* | | Estimate | *SE* | Estimate | *SE* | | Estimate | *SE* |
| **Factor loadings** | |  | |  |  |  |  |  |  |  |  |
| WEMWBS 1 | 0.836 | | 0.035 | | 0.824 | 0.036 | 0.756 | 0.040 | | 0.800 | 0.038 |
| WEMWBS 2 | 0.744 | | 0.037 | | 0.765 | 0.038 | 0.731 | 0.039 | | 0.743 | 0.041 |
| WEMWBS 3 | 0.685 | | 0.037 | | 0.743 | 0.035 | 0.645 | 0.038 | | 0.716 | 0.037 |
| WEMWBS 4 | 0.489 | | 0.039 | | 0.538 | 0.040 |  |  | |  |  |
| WEMWBS 5 | 0.768 | | 0.031 | | 0.820 | 0.033 |  |  | |  |  |
| WEMWBS 6 | 0.527 | | 0.028 | | 0.600 | 0.041 | 0.585 | 0.028 | | 0.628 | 0.042 |
| WEMWBS 7 | 0.375 | | 0.033 | | 0.528 | 0.043 | 0.445 | 0.035 | | 0.586 | 0.045 |
| WEMWBS 8 | 0.700 | | 0.029 | | 0.761 | 0.033 |  |  | |  |  |
| WEMWBS 9 | 0.689 | | 0.038 | | 0.755 | 0.037 | 0.660 | 0.039 | | 0.716 | 0.040 |
| WEMWBS 10 | 0.789 | | 0.029 | | 0.812 | 0.033 |  |  | |  |  |
| WEMWBS 11 | 0.447 | | 0.033 | | 0.491 | 0.042 | 0.522 | 0.034 | | 0.541 | 0.042 |
| WEMWBS 12 | 0.667 | | 0.039 | | 0.797 | 0.042 |  |  |  | |  |
| WEMWBS 13 | 0.558 | | 0.038 | | 0.648 | 0.038 |  |  |  | |  |
| WEMWBS 14 | 0.752 | | 0.031 | | 0.771 | 0.035 |  |  |  | |  |
| **Intercepts** |  | |  | |  |  |  |  |  | |  |
| WEMWBS 1 | 3.514 | | 0.044 | | 3.402 | 0.046 | 3.514 | 0.044 | 3.402 | | 0.046 |
| WEMWBS 2 | 3.750 | | 0.042 | | 3.611 | 0.043 | 3.750 | 0.042 | 3.611 | | 0.043 |
| WEMWBS 3 | 3.748 | | 0.038 | | 3.449 | 0.040 | 3.748 | 0.038 | 3.449 | | 0.040 |
| WEMWBS 4 | 3.821 | | 0.038 | | 3.648 | 0.039 |  |  |  | |  |
| WEMWBS 5 | 3.450 | | 0.040 | | 3.311 | 0.043 |  |  |  | |  |
| WEMWBS 6 | 4.055 | | 0.033 | | 3.805 | 0.037 | 4.055 | 0.033 | 3.805 | | 0.037 |
| WEMWBS 7 | 4.476 | | 0.029 | | 4.095 | 0.038 | 4.476 | 0.029 | 4.095 | | 0.038 |
| WEMWBS 8 | 3.853 | | 0.036 | | 3.613 | 0.039 |  |  |  | |  |
| WEMWBS 9 | 3.652 | | 0.044 | | 3.507 | 0.044 | 3.652 | 0.044 | 3.507 | | 0.044 |
| WEMWBS 10 | 3.729 | | 0.039 | | 3.565 | 0.041 |  |  |  | |  |
| WEMWBS 11 | 4.388 | | 0.032 | | 4.097 | 0.038 | 4.388 | 0.032 | 4.097 | | 0.038 |
| WEMWBS 12 | 3.831 | | 0.045 | | 3.671 | 0.047 |  |  |  | |  |
| WEMWBS 13 | 3.759 | | 0.041 | | 3.455 | 0.042 |  |  |  | |  |
| WEMWBS 14 | 3.540 | | 0.039 | | 3.511 | 0.040 |  |  |  | |  |
| **Residual variances** | | |  | |  |  |  |  |  | |  |
| WEMWBS 1 | 0.335 | | 0.025 | | 0.394 | 0.034 | 0.461 | 0.037 | 0.432 | | 0.040 |
| WEMWBS 2 | 0.373 | | 0.037 | | 0.353 | 0.028 | 0.392 | 0.043 | 0.385 | | 0.036 |
| WEMWBS 3 | 0.296 | | 0.025 | | 0.291 | 0.023 | 0.349 | 0.033 | 0.331 | | 0.028 |
| WEMWBS 4 | 0.522 | | 0.038 | | 0.515 | 0.037 |  |  |  | |  |
| WEMWBS 5 | 0.264 | | 0.019 | | 0.273 | 0.020 |  |  |  | |  |
| WEMWBS 6 | 0.286 | | 0.031 | | 0.339 | 0.029 | 0.222 | 0.030 | 0.304 | | 0.030 |
| WEMWBS 7 | 0.301 | | 0.020 | | 0.461 | 0.033 | 0.244 | 0.019 | 0.396 | | 0.037 |
| WEMWBS 8 | 0.193 | | 0.016 | | 0.192 | 0.019 |  |  |  | |  |
| WEMWBS 9 | 0.565 | | 0.048 | | 0.430 | 0.032 | 0.604 | 0.055 | 0.488 | | 0.039 |
| WEMWBS 10 | 0.193 | | 0.019 | | 0.190 | 0.017 |  |  |  | |  |
| WEMWBS 11 | 0.354 | | 0.026 | | 0.523 | 0.039 | 0.281 | 0.024 | 0.471 | | 0.040 |
| WEMWBS 12 | 0.626 | | 0.049 | | 0.486 | 0.037 |  |  |  | |  |
| WEMWBS 13 | 0.580 | | 0.047 | | 0.490 | 0.036 |  |  |  | |  |
| WEMWBS 14 | 0.248 | | 0.020 | | 0.216 | 0.019 |  |  |  | |  |

**Table S2.2. Results from measurement invariance analyses for configural models of three-factor-solutions separated by age**

|  | WEMWBS  < 50 y. | | WEMWBS  >= 50 y. | | SWEMWBS  < 50 y. | | SWEMWBS  >= 50 y. | |
| --- | --- | --- | --- | --- | --- | --- | --- | --- |
|  | Estimate | *SE* | Estimate | *SE* | Estimate | *SE* | Estimate | *SE* |
| **Factor loadings** | |  |  |  |  |  |  |  |
| Eudaimonic ~ | |  |  |  |  |  |  |  |
| WEMWBS 6 | 0.601 | 0.027 | 0.661 | 0.039 | 0.598 | 0.030 | 0.660 | 0.045 |
| WEMWBS 7 | 0.492 | 0.033 | 0.612 | 0.044 | 0.505 | 0.034 | 0.663 | 0.045 |
| WEMWBS 11 | 0.571 | 0.034 | 0.578 | 0.043 | 0.590 | 0.034 | 0.623 | 0.042 |
| WEMWBS 13 | 0.562 | 0.039 | 0.640 | 0.043 |  |  |  |  |
| Hedonic ~ | |  |  |  |  |  |  |  |
| WEMWBS 1 | 0.846 | 0.034 | 0.827 | 0.036 | 0.833 | 0.039 | 0.825 | 0.039 |
| WEMWBS 3 | 0.697 | 0.037 | 0.749 | 0.035 | 0.682 | 0.039 | 0.721 | 0.038 |
| WEMWBS 5 | 0.776 | 0.031 | 0.825 | 0.033 |  |  |  |  |
| WEMWBS 8 | 0.712 | 0.029 | 0.763 | 0.033 |  |  |  |  |
| WEMWBS 10 | 0.792 | 0.030 | 0.817 | 0.033 |  |  |  |  |
| WEMWBS 14 | 0.764 | 0.030 | 0.778 | 0.035 |  |  |  |  |
| Interpersonal  Relationships ~ | |  |  |  |  |  |  |  |
| WEMWBS 2 | 0.789 | 0.040 | 0.770 | 0.039 | 0.820 | 0.041 | 0.759 | 0.044 |
| WEMWBS 4 | 0.546 | 0.041 | 0.571 | 0.041 |  |  |  |  |
| WEMWBS 9 | 0.794 | 0.039 | 0.809 | 0.038 | 0.730 | 0.039 | 0.756 | 0.040 |
| WEMWBS 12 | 0.734 | 0.041 | 0.854 | 0.043 |  |  |  |  |
| **Intercepts** |  |  |  |  |  |  |  |  |
| WEMWBS 6 | 4.055 | 0.033 | 3.805 | 0.037 | 4.055 | 0.033 | 3.805 | 0.037 |
| WEMWBS 7 | 4.476 | 0.029 | 4.095 | 0.038 | 4.476 | 0.029 | 4.095 | 0.038 |
| WEMWBS 11 | 4.388 | 0.032 | 4.097 | 0.038 | 4.388 | 0.032 | 4.097 | 0.038 |
| WEMWBS 13 | 3.759 | 0.041 | 3.455 | 0.042 |  |  |  |  |
| WEMWBS 1 | 3.514 | 0.044 | 3.402 | 0.046 | 3.514 | 0.044 | 3.402 | 0.046 |
| WEMWBS 3 | 3.748 | 0.038 | 3.449 | 0.040 | 3.748 | 0.038 | 3.449 | 0.040 |
| WEMWBS 5 | 3.450 | 0.040 | 3.311 | 0.043 |  |  |  |  |
| WEMWBS 8 | 3.853 | 0.036 | 3.613 | 0.039 |  |  |  |  |
| WEMWBS 10 | 3.729 | 0.039 | 3.565 | 0.041 |  |  |  |  |
| WEMWBS 14 | 3.540 | 0.039 | 3.511 | 0.040 |  |  |  |  |
| WEMWBS 2 | 3.750 | 0.042 | 3.611 | 0.043 | 3.750 | 0.042 | 3.611 | 0.043 |
| WEMWBS 4 | 3.821 | 0.038 | 3.648 | 0.039 |  |  |  |  |
| WEMWBS 9 | 3.652 | 0.044 | 3.507 | 0.044 | 3.652 | 0.044 | 3.507 | 0.044 |
| WEMWBS 12 | 3.831 | 0.045 | 3.671 | 0.047 |  |  |  |  |
| **Covariances** |  |  |  |  |  |  |  |  |
| Eudaimonic ~ | |  |  |  |  |  |  |  |
| Hedonic | 0.775 | 0.031 | 0.882 | 0.025 | 0.772 | 0.044 | 0.834 | 0.039 |
| Interpersonal Relationships | 0.776 | 0.036 | 0.839 | 0.035 | 0.748 | 0.041 | 0.800 | 0.047 |
| Hedonic ~ |  |  |  |  |  |  |  |  |
| Interpersonal Relationships | 0.859 | 0.022 | 0.929 | 0.016 | 0.854 | 0.034 | 0.989 | 0.030 |
| **Residual variances** | |  |  |  |  |  |  |  |
| WEMWBS 6 | 0.202 | 0.031 | 0.262 | 0.030 | 0.206 | 0.035 | 0.263 | 0.034 |
| WEMWBS 7 | 0.199 | 0.018 | 0.365 | 0.038 | 0.187 | 0.019 | 0.300 | 0.042 |
| WEMWBS 11 | 0.227 | 0.024 | 0.430 | 0.042 | 0.205 | 0.025 | 0.376 | 0.044 |
| WEMWBS 13 | 0.575 | 0.049 | 0.500 | 0.043 |  |  |  |  |
| WEMWBS 1 | 0.318 | 0.025 | 0.389 | 0.035 | 0.339 | 0.038 | 0.392 | 0.045 |
| WEMWBS 3 | 0.279 | 0.026 | 0.282 | 0.023 | 0.299 | 0.032 | 0.323 | 0.030 |
| WEMWBS 5 | 0.252 | 0.020 | 0.266 | 0.020 |  |  |  |  |
| WEMWBS 8 | 0.176 | 0.015 | 0.189 | 0.019 |  |  |  |  |
| WEMWBS 10 | 0.188 | 0.020 | 0.181 | 0.017 |  |  |  |  |
| WEMWBS 14 | 0.229 | 0.018 | 0.206 | 0.020 |  |  |  |  |
| WEMWBS 2 | 0.304 | 0.044 | 0.344 | 0.031 | 0.254 | 0.051 | 0.361 | 0.043 |
| WEMWBS 4 | 0.463 | 0.039 | 0.479 | 0.037 |  |  |  |  |
| WEMWBS 9 | 0.411 | 0.043 | 0.346 | 0.034 | 0.508 | 0.051 | 0.428 | 0.040 |
| WEMWBS 12 | 0.532 | 0.048 | 0.393 | 0.038 |  |  |  |  |

**Table S2.3. Results from measurement invariance analyses for the configural models of bifactor-factor-solutions separated by age**

|  | WEMWBS  < 50 y. | | WEMWBS  >= 50 y. | | SWEMWBS  < 50 y. | | SWEMWBS  >= 50 y. | |
| --- | --- | --- | --- | --- | --- | --- | --- | --- |
|  | Estimate | *SE* | Estimate | *SE* | Estimate | *SE* | Estimate | *SE* |
| **Factor loadings** | |  |  |  |  |  |  |  |
| Well-being ~ |  |  |  |  |  |  |  |  |
| WEMWBS 1 | 1.274 | 0.272 | 1.219 | 0.102 | 1.136 | 0.110 | 0.987 | 0.094 |
| WEMWBS 2 | 1.223 | 0.191 | 1.164 | 0.083 | 1.114 | 0.097 | 0.975 | 0.078 |
| WEMWBS 3 | 1.094 | 0.208 | 1.111 | 0.077 | 1.002 | 0.081 | 0.950 | 0.064 |
| WEMWBS 4 | 0.838 | 0.079 | 0.805 | 0.069 |  |  |  |  |
| WEMWBS 5 | 1.167 | 0.238 | 1.206 | 0.092 |  |  |  |  |
| WEMWBS 6 | 1.000 |  | 1.000 |  | 1.000 |  | 1.000 |  |
| WEMWBS 7 | 0.785 | 0.087 | 0.971 | 0.071 | 0.776 | 0.071 | 0.884 | 0.072 |
| WEMWBS 8 | 1.082 | 0.219 | 1.157 | 0.074 |  |  |  |  |
| WEMWBS 9 | 1.014 | 0.161 | 1.050 | 0.105 | 0.967 | 0.101 | 0.860 | 0.079 |
| WEMWBS 10 | 1.247 | 0.230 | 1.208 | 0.092 |  |  |  |  |
| WEMWBS 11 | 0.952 | 0.072 | 0.926 | 0.071 | 0.904 | 0.076 | 0.818 | 0.085 |
| WEMWBS 12 | 1.005 | 0.264 | 1.161 | 0.104 |  |  |  |  |
| WEMWBS 13 | 0.993 | 0.082 | 0.990 | 0.067 |  |  |  |  |
| WEMWBS 14 | 1.114 | 0.265 | 1.117 | 0.097 |  |  |  |  |
| Eudaimonic ~ | |  |  |  |  |  |  |  |
| WEMWBS 6 |  |  |  |  |  |  |  |  |
| WEMWBS 7 | 1.000 |  | 1.000 |  | 1.181 | 0.624 | 0.241 | 1.319 |
| WEMWBS 11 | 1.385 | 0.861 | 1.142 | 0.403 | 1.000 |  | 1.000 |  |
| WEMWBS 13 | -0.392 | 0.921 | -0.619 | 0.328 |  |  |  |  |
| Hedonic ~ | |  |  |  |  |  |  |  |
| WEMWBS 1 | 1.000 |  | 1.000 |  | 1.000 |  | 1.000 |  |
| WEMWBS 3 | 0.683 | 0.226 | 0.880 | 0.312 | 0.278 | 0.151 | 0.580 | 0.160 |
| WEMWBS 5 | 0.943 | 0.168 | 1.127 | 0.369 |  |  |  |  |
| WEMWBS 8 | 0.820 | 0.165 | 0.705 | 0.232 |  |  |  |  |
| WEMWBS 10 | 0.770 | 0.123 | 0.973 | 0.167 |  |  |  |  |
| WEMWBS 14 | 1.042 | 0.167 | 1.207 | 0.330 |  |  |  |  |
| Interpersonal  Relationships ~ | |  |  |  |  |  |  |  |
| WEMWBS 2 | 1.000 |  | 1.000 |  | 1.000 |  | 1.000 |  |
| WEMWBS 4 | 1.152 | 1.418 | 1.643 | 1.023 |  |  |  |  |
| WEMWBS 9 | 2.968 | 4.773 | 3.821 | 2.611 | 1.000 | 0.278 | 1.649 | 0.426 |
| WEMWBS 12 | 1.707 | 0.525 | 2.821 | 1.443 |  |  |  |  |
| **Intercepts** |  |  |  |  |  |  |  |  |
| WEMWBS 6 | 0.000 |  | 0.000 |  | 0.000 |  | 0.000 |  |
| WEMWBS 7 | 0.000 |  | 0.000 |  | 0.477 | 0.599 | 0.495 | 1.338 |
| WEMWBS 11 | -1.265 | 1.386 | 0.117 | 0.444 | 0.000 |  | 0.000 |  |
| WEMWBS 13 | 0.238 | 1.285 | -0.066 | 0.380 |  |  |  |  |
| WEMWBS 1 | 0.000 |  | 0.000 |  | 0.000 |  | 0.000 |  |
| WEMWBS 3 | 0.439 | 0.606 | 0.309 | 0.522 | -0.010 | 0.323 | 0.037 | 0.231 |
| WEMWBS 5 | 0.277 | 0.461 | 0.118 | 0.573 |  |  |  |  |
| WEMWBS 8 | 0.822 | 0.426 | 0.082 | 0.380 |  |  |  |  |
| WEMWBS 10 | -0.052 | 0.335 | 0.172 | 0.313 |  |  |  |  |
| WEMWBS 14 | 0.747 | 0.456 | 0.755 | 0.538 |  |  |  |  |
| WEMWBS 2 | 0.000 |  | 0.000 |  | 0.000 |  | 0.000 |  |
| WEMWBS 4 | 1.819 | 2.397 | 1.931 | 1.121 |  |  |  |  |
| WEMWBS 9 | 3.135 | 7.370 | 2.643 | 2.696 | 0.497 | 0.398 | 0.398 | 0.408 |
| WEMWBS 12 | 1.826 | 0.989 | 1.563 | 1.568 |  |  |  |  |
| **Covariances** |  |  |  |  |  |  |  |  |
| Well-being ~~ |  |  |  |  |  |  |  |  |
| Eudaimonic | 0.000 |  | 0.000 |  | 0.000 |  | 0.000 |  |
| Hedonic | 0.000 |  | 0.000 |  | 0.000 |  | 0.000 |  |
| Interpersonal relationships | 0.000 |  | 0.000 |  | 0.000 |  | 0.000 |  |
| Eudaimonic ~ | |  |  |  |  |  |  |  |
| Hedonic | -0.055 | 0.049 | -0.063 | 0.025 | -0.050 | 0.034 | -0.000 | 0.060 |
| Interpersonal Relationships | -0.009 | 0.025 | -0.020 | 0.015 | -0.028 | 0.022 | -0.014 | 0.027 |
| Hedonic ~ |  |  |  |  |  |  |  |  |
| Interpersonal Relationships | 0.039 | 0.110 | 0.023 | 0.031 | 0.138 | 0.043 | 0.151 | 0.060 |
| **Residual variances** | |  |  |  |  |  |  |  |
| WEMWBS 6 | 0.224 | 0.049 | 0.291 | 0.033 | 0.193 | 0.036 | 0.224 | 0.033 |
| WEMWBS 7 | 0.176 | 0.049 | 0.267 | 0.054 | 0.134 | 0.053 | 0.339 | 0.168 |
| WEMWBS 11 | 0.138 | 0.081 | 0.300 | 0.070 | 0.191 | 0.046 | -0.065 | 2.780 |
| WEMWBS 13 | 0.547 | 0.073 | 0.476 | 0.043 |  |  |  |  |
| WEMWBS 1 | 0.319 | 0.028 | 0.392 | 0.037 | 0.028 | 0.257 | 0.352 | 0.077 |
| WEMWBS 3 | 0.282 | 0.025 | 0.282 | 0.024 | 0.352 | 0.036 | 0.327 | 0.029 |
| WEMWBS 5 | 0.246 | 0.024 | 0.258 | 0.022 |  |  |  |  |
| WEMWBS 8 | 0.176 | 0.015 | 0.189 | 0.019 |  |  |  |  |
| WEMWBS 10 | 0.191 | 0.020 | 0.184 | 0.017 |  |  |  |  |
| WEMWBS 14 | 0.215 | 0.020 | 0.194 | 0.022 |  |  |  |  |
| WEMWBS 2 | 0.355 | 0.060 | 0.361 | 0.030 | 0.267 | 0.062 | 0.379 | 0.038 |
| WEMWBS 4 | 0.440 | 0.038 | 0.475 | 0.037 |  |  |  |  |
| WEMWBS 9 | 0.142 | 0.596 | 0.198 | 0.076 | 0.495 | 0.062 | 0.359 | 0.061 |
| WEMWBS 12 | 0.547 | 0.182 | 0.380 | 0.042 |  |  |  |  |

**Table S2.4. Results from measurement invariance analyses for the most constrained models (loadings, intercepts) of one-factor solutions separated by age**

|  | WEMWBS  < 50 y. | | | | WEMWBS  >= 50 y. | | SWEMWBS  < 50 y. | | | SWEMWBS  >= 50 y. | |
| --- | --- | --- | --- | --- | --- | --- | --- | --- | --- | --- | --- |
|  | Estimate | | *SE* | | Estimate | SE | Estimate | SE | | Estimate | SE |
| **Factor loadings** | |  | |  |  |  |  |  |  |  |  |
| WEMWBS 1 | 0.794 | | 0.031 | | 0.794 | 0.031 | 0.722 | 0.035 | | 0.722 | 0.035 |
| WEMWBS 2 | 0.724 | | 0.029 | | 0.724 | 0.029 | 0.687 | 0.034 | | 0.687 | 0.034 |
| WEMWBS 3 | 0.696 | | 0.028 | | 0.696 | 0.028 | 0.654 | 0.030 | | 0.654 | 0.030 |
| WEMWBS 4 | 0.499 | | 0.027 | | 0.499 | 0.027 |  |  | |  |  |
| WEMWBS 5 | 0.761 | | 0.028 | | 0.761 | 0.028 |  |  | |  |  |
| WEMWBS 6 | 0.550 | | 0.024 | | 0.550 | 0.024 | 0.585 | 0.025 | | 0.585 | 0.025 |
| WEMWBS 7 | 0.440 | | 0.026 | | 0.440 | 0.026 | 0.501 | 0.027 | | 0.501 | 0.027 |
| WEMWBS 8 | 0.707 | | 0.025 | | 0.707 | 0.025 |  |  | |  |  |
| WEMWBS 9 | 0.696 | | 0.029 | | 0.696 | 0.029 | 0.644 | 0.033 | | 0.644 | 0.033 |
| WEMWBS 10 | 0.768 | | 0.027 | | 0.768 | 0.027 |  |  | |  |  |
| WEMWBS 11 | 0.463 | | 0.026 | | 0.463 | 0.026 | 0.523 | 0.027 | | 0.523 | 0.027 |
| WEMWBS 12 | 0.709 | | 0.030 | | 0.709 | 0.030 |  |  |  | |  |
| WEMWBS 13 | 0.594 | | 0.027 | | 0.594 | 0.027 |  |  |  | |  |
| WEMWBS 14 | 0.721 | | 0.028 | | 0.721 | 0.028 |  |  |  | |  |
| **Intercepts** |  | |  | |  |  |  |  |  | |  |
| WEMWBS 1 | 3.561 | | 0.040 | | 3.561 | 0.040 | 3.598 | 0.040 | 3.598 | | 0.040 |
| WEMWBS 2 | 3.778 | | 0.037 | | 3.778 | 0.037 | 3.812 | 0.037 | 3.812 | | 0.037 |
| WEMWBS 3 | 3.692 | | 0.035 | | 3.692 | 0.035 | 3.722 | 0.035 | 3.722 | | 0.035 |
| WEMWBS 4 | 3.802 | | 0.031 | | 3.802 | 0.031 |  |  |  | |  |
| WEMWBS 5 | 3.482 | | 0.037 | | 3.482 | 0.037 |  |  |  | |  |
| WEMWBS 6 | 4.009 | | 0.030 | | 4.009 | 0.030 | 4.043 | 0.031 | 4.043 | | 0.031 |
| WEMWBS 7 | 4.377 | | 0.028 | | 4.377 | 0.028 | 4.408 | 0.028 | 4.408 | | 0.028 |
| WEMWBS 8 | 3.829 | | 0.034 | | 3.829 | 0.034 |  |  |  | |  |
| WEMWBS 9 | 3.675 | | 0.038 | | 3.675 | 0.038 | 3.706 | 0.037 | 3.706 | | 0.037 |
| WEMWBS 10 | 3.750 | | 0.037 | | 3.750 | 0.037 |  |  |  | |  |
| WEMWBS 11 | 4.323 | | 0.029 | | 4.323 | 0.029 | 4.355 | 0.030 | 4.355 | | 0.030 |
| WEMWBS 12 | 3.848 | | 0.038 | | 3.848 | 0.038 |  |  |  | |  |
| WEMWBS 13 | 3.682 | | 0.035 | | 3.682 | 0.035 |  |  |  | |  |
| WEMWBS 14 | 3.628 | | 0.036 | | 3.628 | 0.036 |  |  |  | |  |
| **Residual variances** | | |  | |  |  |  |  |  | |  |
| WEMWBS 1 | 0.346 | | 0.026 | | 0.395 | 0.034 | 0.486 | 0.038 | 0.442 | | 0.039 |
| WEMWBS 2 | 0.374 | | 0.036 | | 0.353 | 0.028 | 0.415 | 0.043 | 0.388 | | 0.035 |
| WEMWBS 3 | 0.299 | | 0.026 | | 0.295 | 0.024 | 0.350 | 0.033 | 0.331 | | 0.028 |
| WEMWBS 4 | 0.520 | | 0.038 | | 0.516 | 0.037 |  |  |  | |  |
| WEMWBS 5 | 0.267 | | 0.020 | | 0.274 | 0.020 |  |  |  | |  |
| WEMWBS 6 | 0.285 | | 0.031 | | 0.344 | 0.029 | 0.218 | 0.029 | 0.302 | | 0.029 |
| WEMWBS 7 | 0.311 | | 0.021 | | 0.494 | 0.035 | 0.241 | 0.019 | 0.418 | | 0.036 |
| WEMWBS 8 | 0.194 | | 0.016 | | 0.194 | 0.019 |  |  |  | |  |
| WEMWBS 9 | 0.564 | | 0.048 | | 0.431 | 0.031 | 0.619 | 0.057 | 0.493 | | 0.038 |
| WEMWBS 10 | 0.198 | | 0.019 | | 0.188 | 0.017 |  |  |  | |  |
| WEMWBS 11 | 0.355 | | 0.026 | | 0.535 | 0.041 | 0.275 | 0.023 | 0.474 | | 0.040 |
| WEMWBS 12 | 0.626 | | 0.049 | | 0.490 | 0.037 |  |  |  | |  |
| WEMWBS 13 | 0.584 | | 0.047 | | 0.495 | 0.037 |  |  |  | |  |
| WEMWBS 14 | 0.263 | | 0.020 | | 0.222 | 0.020 |  |  |  | |  |

**Table S.2.5. Results from measurement invariance analyses for the most constrained models (loadings, intercepts) of three-factor-solutions separated by age**

|  | WEMWBS  < 50 y. | | WEMWBS  >= 50 y. | | SWEMWBS  < 50 y. | | SWEMWBS  >= 50 y. | |
| --- | --- | --- | --- | --- | --- | --- | --- | --- |
|  | Estimate | *SE* | Estimate | *SE* | Estimate | *SE* | Estimate | *SE* |
| **Factor loadings** | |  |  |  |  |  |  |  |
| Eudaimonic ~ | |  |  |  |  |  |  |  |
| WEMWBS 6 | 0.585 | 0.027 | 0.585 | 0.027 | 0.574 | 0.030 | 0.574 | 0.030 |
| WEMWBS 7 | 0.527 | 0.027 | 0.527 | 0.027 | 0.547 | 0.027 | 0.547 | 0.027 |
| WEMWBS 11 | 0.549 | 0.029 | 0.549 | 0.029 | 0.568 | 0.030 | 0.568 | 0.030 |
| WEMWBS 13 | 0.568 | 0.032 | 0.568 | 0.032 |  |  |  |  |
| Hedonic ~ | |  |  |  |  |  |  |  |
| WEMWBS 1 | 0.818 | 0.032 | 0.818 | 0.032 | 0.807 | 0.039 | 0.807 | 0.039 |
| WEMWBS 3 | 0.716 | 0.028 | 0.716 | 0.028 | 0.703 | 0.033 | 0.703 | 0.033 |
| WEMWBS 5 | 0.783 | 0.028 | 0.783 | 0.028 |  |  |  |  |
| WEMWBS 8 | 0.727 | 0.025 | 0.727 | 0.025 |  |  |  |  |
| WEMWBS 10 | 0.789 | 0.027 | 0.789 | 0.027 |  |  |  |  |
| WEMWBS 14 | 0.747 | 0.028 | 0.747 | 0.028 |  |  |  |  |
| Interpersonal  Relationships ~ | |  |  |  |  |  |  |  |
| WEMWBS 2 | 0.764 | 0.033 | 0.764 | 0.033 | 0.800 | 0.037 | 0.800 | 0.037 |
| WEMWBS 4 | 0.550 | 0.029 | 0.550 | 0.029 |  |  |  |  |
| WEMWBS 9 | 0.784 | 0.032 | 0.784 | 0.032 | 0.757 | 0.033 | 0.757 | 0.033 |
| WEMWBS 12 | 0.785 | 0.032 | 0.785 | 0.032 |  |  |  |  |
| **Intercepts** |  |  |  |  |  |  |  |  |
| WEMWBS 6 | 4.084 | 0.031 | 4.084 | 0.031 | 4.083 | 0.031 | 4.083 | 0.031 |
| WEMWBS 7 | 4.444 | 0.028 | 4.444 | 0.028 | 4.447 | 0.028 | 4.447 | 0.028 |
| WEMWBS 11 | 4.390 | 0.030 | 4.390 | 0.030 | 4.394 | 0.030 | 4.394 | 0.030 |
| WEMWBS 13 | 3.761 | 0.036 | 3.761 | 0.036 |  |  |  |  |
| WEMWBS 1 | 3.543 | 0.041 | 3.543 | 0.041 | 3.566 | 0.042 | 3.566 | 0.042 |
| WEMWBS 3 | 3.677 | 0.036 | 3.677 | 0.036 | 3.699 | 0.038 | 3.699 | 0.038 |
| WEMWBS 5 | 3.464 | 0.038 | 3.464 | 0.038 |  |  |  |  |
| WEMWBS 8 | 3.814 | 0.035 | 3.814 | 0.035 |  |  |  |  |
| WEMWBS 10 | 3.731 | 0.037 | 3.731 | 0.037 |  |  |  |  |
| WEMWBS 14 | 3.609 | 0.036 | 3.609 | 0.036 |  |  |  |  |
| WEMWBS 2 | 3.758 | 0.039 | 3.758 | 0.039 | 3.752 | 0.040 | 3.752 | 0.040 |
| WEMWBS 4 | 3.791 | 0.033 | 3.791 | 0.033 |  |  |  |  |
| WEMWBS 9 | 3.659 | 0.041 | 3.659 | 0.041 | 3.647 | 0.041 | 3.647 | 0.041 |
| WEMWBS 12 | 3.831 | 0.040 | 3.831 | 0.040 |  |  |  |  |
| **Covariances** |  |  |  |  |  |  |  |  |
| Eudaimonic ~ | |  |  |  |  |  |  |  |
| Hedonic | 0.774 | 0.030 | 1.027 | 0.109 | 0.773 | 0.042 | 0.986 | 0.116 |
| Interpersonal Relationships | 0.772 | 0.035 | 0.980 | 0.109 | 0.744 | 0.041 | 0.898 | 0.109 |
| Hedonic ~ |  |  |  |  |  |  |  |  |
| Interpersonal Relationships | 0.858 | 0.022 | 1.007 | 0.094 | 0.859 | 0.033 | 0.982 | 0.102 |
| **Residual variances** | |  |  |  |  |  |  |  |
| WEMWBS 6 | 0.209 | 0.030 | 0.265 | 0.029 | 0.217 | 0.033 | 0.263 | 0.030 |
| WEMWBS 7 | 0.196 | 0.018 | 0.373 | 0.036 | 0.178 | 0.018 | 0.314 | 0.037 |
| WEMWBS 11 | 0.230 | 0.022 | 0.426 | 0.041 | 0.207 | 0.023 | 0.371 | 0.041 |
| WEMWBS 13 | 0.573 | 0.048 | 0.500 | 0.041 |  |  |  |  |
| WEMWBS 1 | 0.324 | 0.025 | 0.387 | 0.035 | 0.360 | 0.037 | 0.398 | 0.044 |
| WEMWBS 3 | 0.283 | 0.026 | 0.290 | 0.024 | 0.294 | 0.031 | 0.329 | 0.030 |
| WEMWBS 5 | 0.251 | 0.020 | 0.267 | 0.020 |  |  |  |  |
| WEMWBS 8 | 0.176 | 0.015 | 0.193 | 0.019 |  |  |  |  |
| WEMWBS 10 | 0.189 | 0.019 | 0.180 | 0.017 |  |  |  |  |
| WEMWBS 14 | 0.237 | 0.018 | 0.211 | 0.020 |  |  |  |  |
| WEMWBS 2 | 0.313 | 0.041 | 0.339 | 0.030 | 0.270 | 0.047 | 0.355 | 0.042 |
| WEMWBS 4 | 0.464 | 0.038 | 0.481 | 0.037 |  |  |  |  |
| WEMWBS 9 | 0.408 | 0.041 | 0.350 | 0.033 | 0.496 | 0.050 | 0.436 | 0.039 |
| WEMWBS 12 | 0.522 | 0.047 | 0.404 | 0.037 |  |  |  |  |

**Table S2.6. Results from measurement invariance analyses for the most constrained models (loadings, intercepts) of bifactor-solutions separated by age**

|  | WEMWBS  < 50 y. | | WEMWBS  >= 50 y. | | SWEMWBS  < 50 y. | | SWEMWBS  >= 50 y. | |
| --- | --- | --- | --- | --- | --- | --- | --- | --- |
|  | Estimate | *SE* | Estimate | *SE* | Estimate | *SE* | Estimate | *SE* |
| **Factor loadings** | |  |  |  |  |  |  |  |
| Well-being ~ |  |  |  |  |  |  |  |  |
| WEMWBS 1 | 1.247 | 0.093 | 1.247 | 0.093 | 1.016 | 0.086 | 1.016 | 0.086 |
| WEMWBS 2 | 1.158 | 0.062 | 1.158 | 0.062 | 1.006 | 0.075 | 1.006 | 0.075 |
| WEMWBS 3 | 1.153 | 0.074 | 1.153 | 0.074 | 0.984 | 0.073 | 0.984 | 0.073 |
| WEMWBS 4 | 0.823 | 0.046 | 0.823 | 0.046 |  |  |  |  |
| WEMWBS 5 | 1.200 | 0.084 | 1.200 | 0.084 |  |  |  |  |
| WEMWBS 6 | 1.000 |  | 1.000 |  | 1.000 |  | 1.000 |  |
| WEMWBS 7 | 0.908 | 0.048 | 0.908 | 0.048 | 0.804 | 0.063 | 0.804 | 0.063 |
| WEMWBS 8 | 1.153 | 0.073 | 1.153 | 0.073 |  |  |  |  |
| WEMWBS 9 | 1.046 | 0.068 | 1.046 | 0.068 | 0.896 | 0.088 | 0.896 | 0.088 |
| WEMWBS 10 | 1.231 | 0.082 | 1.231 | 0.082 |  |  |  |  |
| WEMWBS 11 | 0.935 | 0.050 | 0.935 | 0.050 | 0.841 | 0.049 | 0.841 | 0.049 |
| WEMWBS 12 | 1.089 | 0.078 | 1.089 | 0.078 |  |  |  |  |
| WEMWBS 13 | 1.009 | 0.047 | 1.009 | 0.047 |  |  |  |  |
| WEMWBS 14 | 1.115 | 0.095 | 1.115 | 0.095 |  |  |  |  |
| Eudaimonic ~ | |  |  |  |  |  |  |  |
| WEMWBS 6 |  |  |  |  |  |  |  |  |
| WEMWBS 7 | 1.000 |  | 1.000 |  | 2.284 | 1.474 | 2.284 | 1.474 |
| WEMWBS 11 | 0.914 | 0.228 | 0.914 | 0.228 | 1.000 |  | 1.000 |  |
| WEMWBS 13 | -0.419 | 0.295 | -0.419 | 0.295 |  |  |  |  |
| Hedonic ~ | |  |  |  |  |  |  |  |
| WEMWBS 1 | 1.000 |  | 1.000 |  | 1.000 |  | 1.000 |  |
| WEMWBS 3 | 0.563 | 0.139 | 0.563 | 0.139 | 0.407 | 0.135 | 0.407 | 0.135 |
| WEMWBS 5 | 0.936 | 0.105 | 0.936 | 0.105 |  |  |  |  |
| WEMWBS 8 | 0.663 | 0.104 | 0.663 | 0.104 |  |  |  |  |
| WEMWBS 10 | 0.815 | 0.069 | 0.815 | 0.069 |  |  |  |  |
| WEMWBS 14 | 1.053 | 0.096 | 1.053 | 0.096 |  |  |  |  |
| Interpersonal  Relationships ~ | |  |  |  |  |  |  |  |
| WEMWBS 2 | 1.000 |  | 1.000 |  | 1.000 |  | 1.000 |  |
| WEMWBS 4 | 1.051 | 0.417 | 1.051 | 0.417 |  |  |  |  |
| WEMWBS 9 | 2.425 | 0.893 | 2.425 | 0.893 | 1.230 | 0.201 | 1.230 | 0.201 |
| WEMWBS 12 | 1.844 | 0.326 | 1.844 | 0.326 |  |  |  |  |
| **Intercepts** |  |  |  |  |  |  |  |  |
| WEMWBS 6 | 0.000 |  | 0.000 |  | 0.000 |  | 0.000 |  |
| WEMWBS 7 | 0.000 |  | 0.000 |  | -1.012 | 1.463 | -1.012 | 1.463 |
| WEMWBS 11 | -0.099 | 0.310 | -0.099 | 0.310 | 0.000 |  | 0.000 |  |
| WEMWBS 13 | -0.053 | 0.327 | -0.053 | 0.327 |  |  |  |  |
| WEMWBS 1 | 0.000 |  | 0.000 |  | 0.000 |  | 0.000 |  |
| WEMWBS 3 | -0.117 | 0.294 | -0.117 | 0.294 | -0.047 | 0.237 | -0.047 | 0.237 |
| WEMWBS 5 | 0.014 | 0.229 | 0.014 | 0.229 |  |  |  |  |
| WEMWBS 8 | 0.161 | 0.217 | 0.161 | 0.217 |  |  |  |  |
| WEMWBS 10 | -0.014 | 0.173 | -0.014 | 0.173 |  |  |  |  |
| WEMWBS 14 | 0.664 | 0.214 | 0.664 | 0.214 |  |  |  |  |
| WEMWBS 2 | 0.000 |  | 0.000 |  | 0.000 |  | 0.000 |  |
| WEMWBS 4 | 1.415 | 0.533 | 1.415 | 0.533 |  |  |  |  |
| WEMWBS 9 | 1.575 | 1.051 | 1.575 | 1.051 | 0.395 | 0.237 | 0.395 | 0.237 |
| WEMWBS 12 | 1.072 | 0.469 | 1.072 | 0.469 |  |  |  |  |
| **Covariances** |  |  |  |  |  |  |  |  |
| Well-being ~~ |  |  |  |  |  |  |  |  |
| Eudaimonic | 0.000 |  | 0.000 |  | 0.000 |  | 0.000 |  |
| Hedonic | 0.000 |  | 0.000 |  | 0.000 |  | 0.000 |  |
| Interpersonal relationships | 0.000 |  | 0.000 |  | 0.000 |  | 0.000 |  |
| Eudaimonic ~ | |  |  |  |  |  |  |  |
| Hedonic | -0.086 | 0.026 | -0.075 | 0.020 | -0.019 | 0.022 | 0.009 | 0.016 |
| Interpersonal Relationships | -0.019 | 0.014 | -0.027 | 0.014 | -0.009 | 0.012 | 0.003 | 0.014 |
| Hedonic ~ |  |  |  |  |  |  |  |  |
| Interpersonal Relationships | 0.050 | 0.036 | 0.046 | 0.032 | 0.156 | 0.038 | 0.187 | 0.046 |
| **Residual variances** | |  |  |  |  |  |  |  |
| WEMWBS 6 | 0.231 | 0.032 | 0.289 | 0.031 | 0.179 | 0.035 | 0.221 | 0.039 |
| WEMWBS 7 | 0.139 | 0.028 | 0.245 | 0.053 | 0.045 | 0.120 | 0.077 | 0.218 |
| WEMWBS 11 | 0.192 | 0.030 | 0.327 | 0.053 | 0.232 | 0.034 | 0.396 | 0.046 |
| WEMWBS 13 | 0.562 | 0.051 | 0.475 | 0.044 |  |  |  |  |
| WEMWBS 1 | 0.316 | 0.027 | 0.387 | 0.036 | 0.166 | 0.117 | 0.298 | 0.098 |
| WEMWBS 3 | 0.284 | 0.025 | 0.287 | 0.023 | 0.338 | 0.032 | 0.335 | 0.028 |
| WEMWBS 5 | 0.245 | 0.021 | 0.261 | 0.020 |  |  |  |  |
| WEMWBS 8 | 0.183 | 0.015 | 0.190 | 0.019 |  |  |  |  |
| WEMWBS 10 | 0.190 | 0.020 | 0.183 | 0.017 |  |  |  |  |
| WEMWBS 14 | 0.216 | 0.019 | 0.196 | 0.021 |  |  |  |  |
| WEMWBS 2 | 0.364 | 0.043 | 0.364 | 0.029 | 0.304 | 0.046 | 0.366 | 0.039 |
| WEMWBS 4 | 0.450 | 0.040 | 0.478 | 0.036 |  |  |  |  |
| WEMWBS 9 | 0.242 | 0.108 | 0.224 | 0.075 | 0.454 | 0.058 | 0.408 | 0.050 |
| WEMWBS 12 | 0.508 | 0.067 | 0.382 | 0.042 |  |  |  |  |
